# Integrating a newly developed BAC-based physical mapping resource for *Lolium perenne* with a genome-wide association study across a *L. perenne* European ecotype collection identifies genomic contexts associated with agriculturally important traits

**DOI:** 10.1093/aob/mcy230

**Published:** 2019-02-02

**Authors:** J Harper, J De Vega, S Swain, D Heavens, D Gasior, A Thomas, C Evans, A Lovatt, S Lister, D Thorogood, L Skøt, M Hegarty, T Blackmore, D Kudrna, S Byrne, T Asp, W Powell, N Fernandez-Fuentes, I Armstead

**Affiliations:** 1 Institute of Biological, Environmental and Rural Sciences, Aberystwyth University, Aberystwyth, UK; 2 Earlham Institute, Norwich Research Park, Norwich, UK; 3 Arizona Genomics Institute, School of Plant Sciences, University of Arizona, Tucson, AZ, USA; 4 Teagasc, Department of Crop Science, Carlow, Ireland; 5 Department of Molecular Biology and Genetics, Crop Genetics and Biotechnology, Aarhus University, Slagelse, Denmark; 6 Scotland’s Rural College, Edinburgh, UK

**Keywords:** *Lolium perenne*, BAC-based physical map, FPC, LTC, GWAS, ecotypes, candidate genes, flowering, heading date, water-soluble carbohydrate, plant width

## Abstract

**Background and Aims:**

*Lolium perenne* (perennial ryegrass) is the most widely cultivated forage and amenity grass species in temperate areas worldwide and there is a need to understand the genetic architectures of key agricultural traits and crop characteristics that deliver wider environmental services. Our aim was to identify genomic regions associated with agriculturally important traits by integrating a bacterial artificial chromosome (BAC)-based physical map with a genome-wide association study (GWAS).

**Methods:**

BAC-based physical maps for *L. perenne* were constructed from ~212 000 high-information-content fingerprints using Fingerprint Contig and Linear Topology Contig software. BAC clones were associated with both BAC-end sequences and a partial minimum tiling path sequence. A panel of 716 *L. perenne* diploid genotypes from 90 European accessions was assessed in the field over 2 years, and genotyped using a *Lolium* Infinium SNP array. The GWAS was carried out using a linear mixed model implemented in TASSEL, and extended genomic regions associated with significant markers were identified through integration with the physical map.

**Key Results:**

Between ~3600 and 7500 physical map contigs were derived, depending on the software and probability thresholds used, and integrated with ~35 k sequenced BAC clones to develop a resource predicted to span the majority of the *L. perenne* genome. From the GWAS, eight different loci were significantly associated with heading date, plant width, plant biomass and water-soluble carbohydrate accumulation, seven of which could be associated with physical map contigs. This allowed the identification of a number of candidate genes.

**Conclusions:**

Combining the physical mapping resource with the GWAS has allowed us to extend the search for candidate genes across larger regions of the *L. perenne* genome and identified a number of interesting gene model annotations. These physical maps will aid in validating future sequence-based assemblies of the *L. perenne* genome.

## INTRODUCTION

*Lolium perenne* (perennial ryegrass) is widely grown in Northern Europe and in temperate areas worldwide as a forage and amenity grass. For a number of decades there have been public and private plant breeding programmes for *L. perenne*, and the *L. perenne* seed constituent is both the largest and most commercially valuable component within many marketed grass seed mixes. Due to this wide cultivation, in addition to its economic and agricultural value, it contributes to the environmental footprint of large areas of grasslands. Thus, the trait biology of this grass can have major effects on landscapes, soil structures, hydrology, water quality and carbon sequestration, in addition to the ‘intended’ qualities that recommend it for on-farm use.

*Lolium perenne* is a diploid species that contains a two-locus gametophytic self-incompatibility system (Thorogood *et al.*, 2002, 2017). As a consequence, both commercial and *in situ* ecotype populations are often highly heterozygous and variable both within and between populations (Blackmore *et al.*, 2015, 2016). One advantage of this is that there is considerable phenotypic variation across the gene pool that is available to plant breeders for targeted crop improvement. The disadvantage is that delimiting and controlling this variation, in order to define a variety that meets the requirements for distinctness, uniformity and stability, can be a major challenge.

Historically, in order to understand the complexity of the genetic control of target traits in *L. perenne* and related species, there has been a major focus on the analysis of quantitative trait loci (QTL) using genetic maps and trait data developed from tightly defined families (for an overview see Shinozuka *et al.*, 2012). These have been derived from two-way pseudo-testcrosses (i.e. a pair cross of two heterozygous individuals; e.g. Inoue *et al.*, 2004; Ergon *et al.*, 2006; Byrne *et al.*, 2009; Brown *et al.*, 2010; Hegarty *et al.*, 2013; Paina *et al.*, 2016) or, where available, using inbred lines or doubled haploid genotypes to derive *F*_2_- and BC_1_-type populations (e.g. Yamada *et al.*, 2004; Turner *et al.*, 2006; Kobayashi *et al.*, 2011; Foito *et al.*, 2015) and *Lolium/Festuca* introgression populations (Humphreys *et al.*, 2005; Moore *et al.*, 2005; Armstead *et al.*, 2006a, b). More recent developments for rapid generation of high-density genetic maps, such as the *Lolium*/*Festuca* DArT array (Kopecky *et al.*, 20 09), a *Lolium* Infinium SNP-genotyping array (Blackmore *et al.*, 2015) and genotyping by sequencing (Ashraf *et al.*, 2016; Pembleton *et al.*, 2016; Velmurugan *et al.*, 2016), have enabled a number of genome-wide association studies (GWAS) and germplasm sampling studies to be conducted across *L. perenne* populations, often in the context of advanced breeding populations, aimed at defining marker/trait associations with greater resolution for both candidate gene identification and genomic selection. Across these studies, associations and genomic predictions have been developed particularly for heading date, but also for crown rust resistance, seed yield, biomass, water-soluble carbohydrate (WSC) and dry matter digestibility (Blackmore *et al.*, 2015, 2016; Fè *et al.*, 2015; Ashraf *et al.*, 2016; Grinberg *et al.*, 2016; Byrne *et al.*, 2017; Arojju *et al.*, 2018; Cericola *et al.*, 2018).

Concurrent with the development of new methods of genetic analysis, there have been remarkable advances in the technologies and software available for generating genome sequences and assembling long-distance contiguous DNA sequence reads, which can, or are approaching the ability to, approximate complete chromosome (pseudomolecule) sequences. In-depth transcriptome sequencing and assembly have also guided detailed annotations of many genomes that both describe gene structure and predict function. Recent published advances in this area include two initial genome assemblies for *L. perenne* (Byrne *et al.*, 2015; Velmurugan *et al.*, 2016) and a high-quality reference genome assembly for *Hordeum vulgare* (Mascher *et al.*, 2017). These have built on the more established reference assemblies for rice (International Rice Genome Sequencing Project, 2005) and *Brachypodium distachyon* (International Brachypodium Initiative, 2010) amongst grass species. Along with direct sequence assembly methods, other approaches that rely on different metrics have been used in developing and validating sequence-based contigs, including bacterial artificial chromosome (BAC)-based physical mapping (e.g. Gu *et al.*, 2009; Zhou *et al.*, 2009; Fleury *et al.*, 2010; Ariyadasa *et al.*, 2014; Varshney *et al.*, 2014). This technique relies on developing ‘fingerprints’ of individual BACs based on restriction enzyme fragment sizes, and identifying overlapping BAC clones through fingerprint matching (Sulston *et al.*, 1988; Luo *et al.*, 2003). Extended regions of contiguity can then be developed using software approaches implemented in Fingerprint Contig (Soderlund *et al.*, 1997, 2000) and Linear Topology Contig (Frenkel *et al.*, 2010). Physical map assemblies can then be integrated with sequence-based assemblies through BAC-end sequencing (BES) and selective BAC sequencing, often based on projecting a minimum tiling path (MTP) of BACs through each physical contig. This has proved to be an important adjunct in constructing and validating sequence scaffolds in genome assemblies (Mascher *et al.*, 2017).

For many decades, plant breeders and others have been collecting perennial ryegrass and related germplasm from diverse locations in order to preserve and curate the range of genetic variation available within this species group; the gene bank at Aberystwyth University alone contains in excess of 6000 different accessions of *Lolium* spp. However, characterizing such accessions genotypically or phenotypically is a major challenge, but is also fundamental to their future exploitation. With climate uncertainty and changing patterns of land use, it is pressing that we develop a greater understanding of the potential of these germplasm collections for both enhancing the agricultural relevance of forage grass varieties and addressing new challenges that may relate more directly to enhancing environmental services, such as water and nutrient use efficiency and carbon sequestration. The work we present here is part of this effort to exploit new germplasm resources through linking new genetic and genomic approaches with comprehensive phenotyping studies. Specifically, the aim of the present study has been to integrate a newly developed genomics resource for *L. perenne* with the results of a GWAS of a broad selection of European *L. perenne* ecotypes in order to define the genomic contexts of significant GWAS markers and to suggest candidate genes. This will enable the prioritization of particular *L. perenne* accessions and genotypes for further evaluation in terms of broadening the range of germplasm available and deepening our understanding of the biology that underpins addressing future challenges.

## MATERIALS AND METHODS

**Fig. 1.**
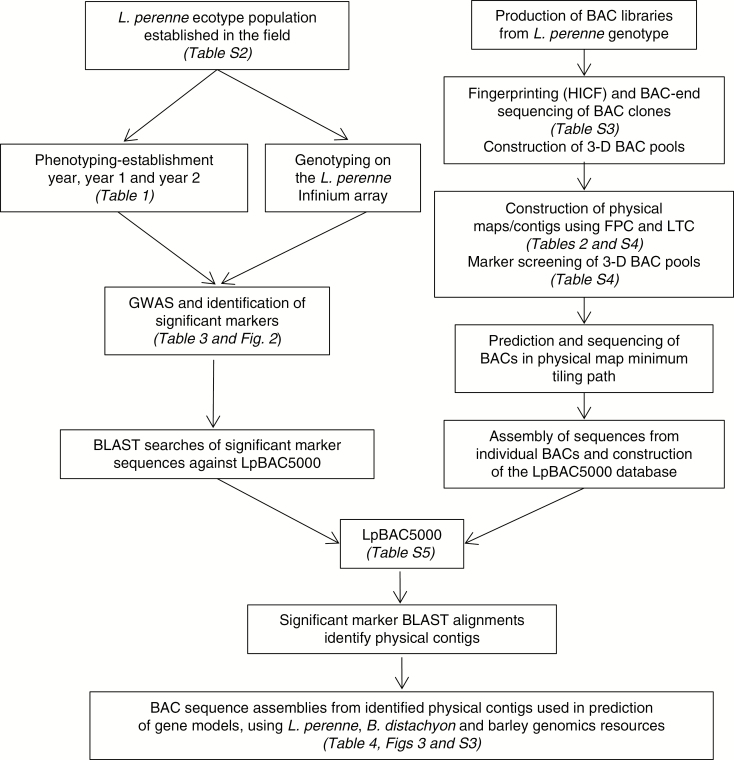
Diagrammatic representation of the integration of the *L. perenne* GWAS with the new sequence-tagged physical mapping resources for the identification of candidate genomic regions for agriculturally significant traits. Related figures and tables are indicated in parentheses. The LpBAC5000 database can be accessed from https://doi.org/10.20391/dfb05330-7485-444f-a475-8310bee5d510 and Supplementary Data Table S4 from https://doi.org/10.20391/bb56e6d7-8913-4bd7-8167-2b7e4c01382b.

Figure 1 illustrates the workflow described in the following sections and the Supplementary Data.

### Development of a BAC-based *L. perenne* physical map

The development of the *L. perenne* BAC-based physical map is described in more detail in Supplementary Data Methods S1. Briefly, two BAC libraries, each consisting of 120 960 clones, were developed from an inbred *L. perenne* genotype using HindIII or BstY1 partial restriction and all clones were fingerprinted using high-information-content fingerprinting (HICF; Luo *et al.*, 2003). Fingerprint data derived from the individual BACs were used to construct physical maps using FingerPrint Contig (FPC) and Linear Topology Contig (LTC) software. We obtained FPC v. 9.4 from http://www.agcol.arizona.edu/software/fpc/, with HICF data analysed largely as described in Kim *et al.* (2007). The LTC software was obtained from MultiQTL Ltd (Haifa, Israel). Initial LTC physical map construction was performed using LTCbeta 2.1 [physical map version LTC-18(2s)] and subsequent LTC physical maps were constructed using LTC 1.4.6. using a range of different stringencies from *P* = 1 × 10^−12^ to 1 × 10^−27^ (physical map versions LTC-12 to LTC-27) for the construction of networks of significant clone overlaps, from which were derived the BAC contigs constituting each version of the physical map.

### Sequence tagging of the *L. perenne* physical map

#### Shotgun clone sequencing.

An MTP was predicted from the physical map version LTC-18(2s), and 39 202 BAC clones were shotgun-sequenced, largely using the methods of Mascher *et al.* (2017). Each BAC was assembled individually using ABySS with Kmer length 71 (v. 1.5.2; Simpson *et al.*, 2009) and BACs with a total assembly length <30 or >200 kb were re-sequenced. Methods employed in the further concatenation of the assemblies, alignment with the *L. perenne* draft genome assembly of Byrne *et al.* (2015) and further characterization of the sequence database used for anchoring GWAS markers to the physical maps (designated the LpBAC5000 database) are provided in Supplementary Data Methods S2.

#### BAC-end sequences.

BAC-end sequencing of all 24 1920 clones was carried out following standard protocols using M13 primers, Sanger sequencing and capillary fragment separation and detection (Kim *et al.*, 2007).

### Three-dimensional BAC pool screening with PCR-based markers

Previously genetically mapped markers were associated with BACs using 3-D BAC pool screening. BAC pools were constructed from both BAC libraries by Amplicon Express (Pullman, WA, USA) according to a ‘superpool/matrix pool’ approach (Bouzidi *et al.*, 2006). PCR-based markers were screened across the BAC pools using either agarose gel electrophoresis or KASP technology (LGC, Teddington, UK). Further details are provided in Supplementary Data Methods S3 [which also describes a restriction site associated DNA (RAD) sequencing pilot study for assigning BES with genetic positions] with additional marker sequences provided in Supplementary Data Table S1.

### Population genotyping and phenotyping

The development and genotyping of the ecotype population has been described previously (Blackmore *et al.*, 2015). Briefly, 716 genotypes from a European ecotype collection sampled from 90 locations (*n* = 8 for 89 locations, *n* = 4 for one location; Supplementary Data Table S2) were maintained and grown as spaced plants in a field site at Aberystwyth, UK, over a period of 2 years. Plants were genotyped using a custom Illumina Infinium iSelect array across 3425 SNPs and 2034 informative markers (minimum minor allele frequency set at 5 %) spanning the *L. perenne* genome identified.

Plants were grown as spaced plants in three replicated blocks, with management of plants following standard procedures for perennial ryegrass national testing (Supplementary Data Methods S4). Plants were evaluated for a range of growth-related phenotypes and biochemical measures as described in Table 1. Field-based measurements and analytical chemistry measurements for WSC, neutral and acid detergent fibre (ADF/NDF), total nitrogen (N) and dry matter digestibility were conducted according to standard practices used in perennial ryegrass national variety assessments (Supplementary Data Methods S5).

**Table 1.** Traits measured for the GWAS trial

Trait	Month	Year	Replications	*n*
Plant establishment	November	Establishment	3	716
Plant width	May	1	3	709
Leaf width	May	1	3	708
Vegetative biomass	June	1	3	705
Heading date	–	1	3	705
Plant regrowth	July	1	3	702
Plant regrowth	September	1	3	692
Stem rust	August	1	3	694
Heading date	–	2	3	682
Spring growth	April	2	3	686
Dry weight	June	1	2	686
Neutral detergent fibre	June	1	2	679
Acid detergent fibre	June	1	2	685
Dry matter digestibility	June	1	2	681
Total nitrogen	June	1	2	677
Water-soluble carbohydrate	June	1	2	682

### GWAS

The GWAS was implemented using TASSEL (Trait Analysis by aSSociation, Evolution, and Linkage) 5.2.4.1 (https://bitbucket.org/tasseladmin/tassel-5-source/downloads/;Bradbury *et al.*, 2007) using the mixed linear model MLM (PCA+K) workflow, in which genetic population structure is estimated using principal components analysis and fitted as a fixed effect, and genotype relatedness is estimated through a kinship matrix and fitted as a random effect (Zhang *et al.*, 2010). TASSEL default parameters were used throughout (the first five principal components were used for population structure estimation; minimum minor allele frequency was set at 5 %). Dry weight data were square-root-transformed prior to analysis. False discovery rates were controlled using the Benjamini–Hochberg procedure at a level of 10 %.

## RESULTS

### Physical map construction

Summary statistics for the two BAC libraries and their subsequent processing are described in Supplementary Data Table S3. The details of the derived physical maps assembled using FPC and LTC are summarized in Table 2. In comparing the different versions of the physical map, LTC-15 and particularly LTC-12 incorporated fewer clones than the other physical maps. The main reason for this was that at the lower stringency levels more clones had >500 significant overlaps and so were excluded from the subsequent assemblies. Both FPC physical maps incorporated more clones than any of the LTC physical maps, presumably as a consequence of the different methods employed by the two softwares for establishing contig reliability. Overall, 29 482 clones were excluded from all of the physical map versions, 125 321 clones were common to all of the LTC physical maps from LTC-18 to LTC-27, and 121 842 of these were also common to FPC1.03. Where clones were incorporated in contigs across cut-off thresholds and assembly methods, contig composition and clone order within contigs tended to be highly conserved (for a list of the physical maps contigs generated, see Supplementary Data Table S4, available from https://doi.org/10.20391/bb56e6d7-8913-4bd7-8167-2b7e4c01382b).

**Table 2.** Summary statistics for *L. perenne* physical maps

Version	FPC-1.02	FPC-1.03	LTC-12	LTC-15	LTC-18	LTC-21	LTC-24	LTC-27	LTC-18(2s)
HICF BACS*	211 880	211 880	211 880	211 880	211 880	211 880	211 880	211 880	154 836
Contigs (ctgs)	7472	4029	3765	3695	4110	4745	5937	6780	4048
Number of BACS in ctgs									
ctgs ≤1000^†^	163 865	163 112	107 280	132 519	145 994	151 793	152 579	147 193	142 887
ctgs >1000^†^	45 052	45 805	–	–	–	–	–	–	–
Singletons	2963	2963	104 600	79 361	65 886	60 087	59 301	64 687	11 949
BACS/ctg									
2–5	1245	468	–	–	–	–	–	–	–
6–10	1646	549	806	638	700	855	1323	1696	712
11–20	1920	738	1028	914	1029	1265	1868	2376	964
21–30	1000	529	677	603	667	824	1082	1286	679
31–50	953	639	727	750	841	958	1037	1033	824
51–100	613	752	471	619	707	716	569	370	692
101–1000	91	349	56	171	165	127	58	19	177

*Number of BACs associated with HICFs.

^†^Total number of physical contigs consisting of either ≤1000 or >1000 BACs.

### Direct anchoring of physical maps to genetic/genomic positions

Of the 1240 markers that were screened on the superpools and matrix pools, 887 could be assigned to contigs (excluding contigs with >1000 constituent BACs) across the FPC and LTC methods and stringencies with the requirement that the marker was positive for a minimum of two different BACs in the same contig. Overall, this resulted in between 522 and 711 contigs being assigned genetic/genomic positions, depending on the assembly software and stringency used. Marker/BAC/physical contig associations are given in Supplementary Data Table S4.

### BAC-end sequences

Summary statistics for the BES of 241920 clones are given in Supplementary Data Table S3 (BES data are available from NCBI under GenBank accession numbers MJ032229–MJ424519 and can also be downloaded from https://doi.org/10.20391/61921116-ddd0-4d85-b0fd-e0d734bc63c8). For the clones that were incorporated in the versions of the physical maps, ~71 % had BES for both ends of the clone, 21 % had BES for one end of the clone and 8 % had no associated BES information.

### Shotgun clone sequencing

We assembled 35 434 BACs individually. The average BAC assembly length was 77.5 kb, the median number of sequences was 9 and N50 contig length was 10.3 ± 4.8 kb (Supplementary Data Fig. S1A, B). From these, 33 480 BACs were extracted with total contig sequence length in the size range 30–200 kb, which formed the basis of the LpBAC5000 database. Further analysis details are provided in Supplementary Data Results S1. This sequencing project files are deposited online under Bioproject PRJNA475227, BioSample:SAMN09382314, SRA Sample:SRS3412769 and SRA Study:SRP150420.

### LpBAC5000 database

Overall, LpBAC5000 consisted of 207 958 sequences totalling ~2634 Mb extracted from 33 480 clones (~85 %) of the predicted MTP. The average contig size was ~12.7 kb and the average sequence length per BAC was ~78.7 kb. In order to estimate the coverage of the gene space within LpBAC5000, the alignment lengths of LpBAC5000 sequence contigs to gene models within the lope_transcripts.V1.0.fasta, Bdistachyon_314_v3.1.cds.fasta and Hv_IBSC_PGSB_r1_CDS_HighConf.fa databases were estimated. For the *L. perenne* database, ~89 % of the gene space was represented, tagging ~97 % of the gene models. For the *B. distachyon* and *H. vulgare* databases, the equivalent figures were ~73 and ~78 % for the gene space and ~74 and ~85 % for the tagged gene models, respectively. Summary statistics of the LpBAC5000 sequence database are given in Supplementary Data Table S5A, B. The LpBAC5000 sequence file can be downloaded from https://doi.org/10.20391/dfb05330-7485-444f-a475-8310bee5d510.

### Coverage of the *L. perenne* genome within the physical map

*Lolium perenne* genome coverages within the physical maps were estimated to range from 1.57–2.11 Gb without correction for MTP BACs with no associated sequences to 1.85–2.5 Gb with correction for missing BAC MTP sequences, depending on the physical map version. This compares with the Byrne *et al.* (2015) estimate of 2.07 Gb from their draft genome assembly (Supplementary Data Table S6).

### GWAS analysis

**Fig. 2.**
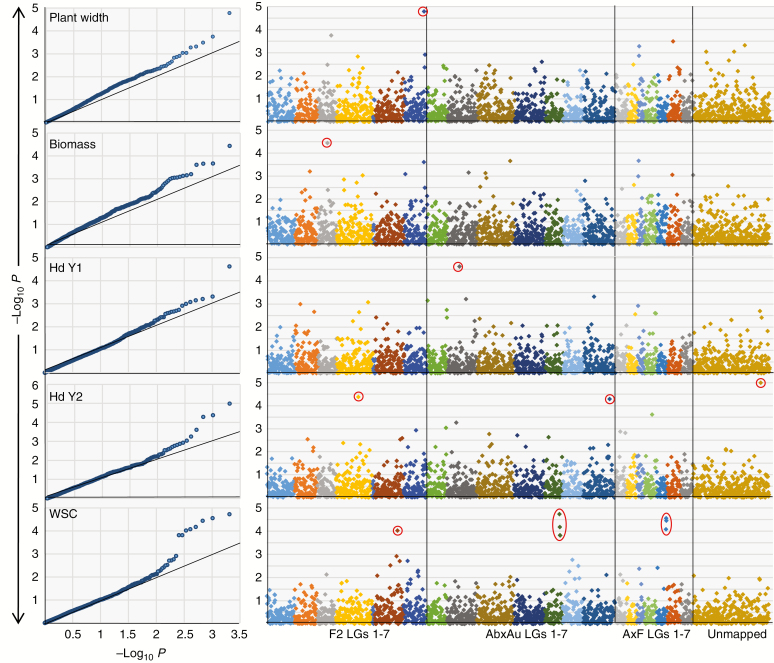
QQ plots (left) and Manhattan plots (right) for the traits for which significant markers were identified using 10 % FDR. Red-circled markers on the Manhattan plots indicate the significant markers. For WSC, all the LG5 red-circled markers were associated with different exons from the same gene (marker contigs ctg35543 and 40624). Hd, heading date; F2, AbxAu and AxF *F*_2_, AberMagic × Aurora and Amenity × Forage perennial ryegrass genetic maps, respectively; Y, year.

The results of the GWAS analysis are summarized in Table 3, Fig. 2 and Supplementary Data Fig. S2. Quantile–quantile (QQ) plots indicated that the distributions of probability estimates were slightly above chance expectation for a number of the field-based measurements of plant biomass and architecture, though not heading date. Measures of plant chemical composition were in line with expectation. For five of the 16 trait scores analysed, a total of 13 significant marker/trait associations, corresponding to eight different genetic map positions, were identified within the false discovery rate (FDR) of 10 % (Fig. 2). Of these, four were associated with heading date (one of which has not been assigned a genetic map position). Seven different markers were associated with WSC; however, four of these were SNPs from within the same marker contig (ctg35543) and a further two were from a marker contig (ctg40624) predicted to be a different exon of the same gene as ctg35543. The seventh marker was a separate locus genetically mapping to a different chromosome. One marker was significantly associated with plant biomass within 10 % FDR. However, the QQ plot for this trait does not indicate a major deviation from expectation for that marker, suggesting this may be a false positive.

**Table 3.** Significant marker/trait associations from the GWAS

					Genetic map position			
					*F*_2_^1^		Ab × Au^2^	
Trait	Year	Month	Marker	*P* ^3^	LG	cM	LG	cM
Plant width	1	May	ctg8394-538	1.66 × 10^−5^	7	68	–	–
Heading date	1	–	ctg50617-428	2.41 × 10^−5^	–	–	2	35
WSC^4,5^	1	June	ctg35543-1175	1.86 × 10^−5^	–	–	5	41
WSC	1	June	ctg41386-226	9.62 × 10^−5^	6	83	6	43
Heading date	2	–	ctg8613-723	1.02 × 10^−5^	–	–	–	–
Plant biomass	1	June	ctg54379-73	3.71 × 10^−5^	3	55	3	50
Heading date	2	–	ctg9479-1216	4.17 × 10^−5^	4	56	4	43
Heading date	2	–	ctg20671-156	5.13 × 10^−5^	–	–	7	50

^1^ *L. perenne F*_2_ genetic map.

^2^ *L. perenne* AberMagic × Aurora genetic map.

^3^Significant with FDR controlled within 10 %.

^4^Markers ctg35543_503/641/365/281 (number after ‘–‘ is the position of the SNP within the contig) were also significant within 10 % FDR (*P* = 2.79 × 10^−5^ to 8.41 × 10^−5^).

^5^Markers ctg40624-321/549 were also significantly associated with WSC (*P* = 1.52 × 10^−4^). Ctg40624 genetically maps to the same position on LG5 and is likely to be derived from a different exon from the same gene as ctg35543.

### Integration of the GWAS with the sequence-tagged physical map

**Fig. 3.**
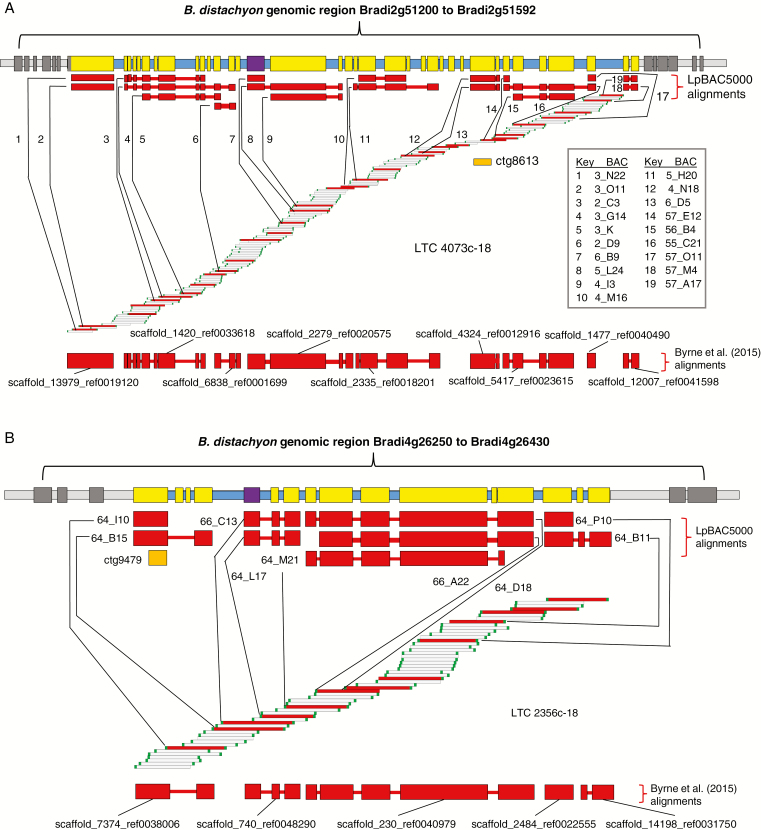
Diagrammatic representations of physical map contigs 4073c-18 (A) and 2356c-18 (B) in relation to conserved syntenic regions in the *B. distachyon* genome. Horizontal bars at the top of each panel represent the genomic region containing the *B. distachyon* gene models indicated. Yellow or purple rectangles on the blue background illustrate the relative positions of the *B. distachyon* gene models within the defined region; the purple rectangles indicate the positions of the candidate genes identified for both physical contigs in Table 4 and discussed in the text. Dark grey rectangles on the light grey background illustrate the positions of *B. distachyon* gene models just outside of the defined region. Wide horizontal red bars illustrate the *B. distachyon* gene models tagged within LpBAC5000 or within the *L. perenne* genomic scaffolds from Byrne *et al.* (2015), above and below the contig illustration, respectively. Narrow horizontal red bars indicate where gene space sequences are contiguous. Orange filled boxes indicate the aligned positions of the marker sequences. Red BACs within the contig illustrations indicate BACs for which sequence information is available. In (A), the BACs from which the aligned LpBAC5000 contigs were derived are number-coded and detailed in the key. In (B) the BAC names are given directly.

BLAST searches of significant marker sequences against the LpBAC5000 database were used to identify associated physical map contigs (Table 4, Fig. 3, Supplementary Data Fig. S3). For six of the identified physical contigs there was a strong indication of conserved synteny, with both *B. distachyon* and *H. vulgare* with physical contigs encompassing between eight and 32 *B. distachyon* gene models. The remaining physical contig, 1969c-18, identified by marker ctg20671, could be associated with three gene models in total; two were physically close on *B. distachyon* chromosome 3 and the third was from chromosome 1. This last gene model had no *H. vulgare* orthologue at the stringencies tested. Marker ctg41386-226 was significantly associated with WSC, but could not be aligned with any of the physical contigs through the LpBAC5000 database.

**Table 4.** *Brachypodium distachyon* (*Bd*) and *Hordeum vulgare* (*Hv*) gene models associated with physical contigs identified by significant markers from the GWAS

*Bd* ^1^	*Hv* ^1^	Functional annotations^1,2^
**Marker = ctg8613 Trait = heading date (Y2) *P* = 1.02 × 10** ^**−5**^ **Physical ctg = 4073c-18**		
2_51255	3_075220/75250	K11838 - ubiquitin carboxyl-terminal hydrolase 7 (USP7, UBP15) (1 of 4)_[RICE]ubiquitin carboxyl-terminal hydrolase, putative, expressed_[ARAB]ubiquitin-specific protease 13
2_51270	3_075270	–
2_51272	3_075260	–
2_51274	3_075270	–
2_51276	3_075260	–
2_51280	3_075310	glycosyl hydrolase (GH), subfamily GH3_[RICE]periplasmic beta-glucosidase precursor, putative, expressed_[ARAB]glycosyl hydrolase family protein
2_51291	–	–
2_51300	3_075340	PTHR10910:SF95 - DOUBLE-STRANDED RNA-BINDING PROTEIN 4 (1 of 1)_[RICE]double-stranded RNA binding motif containing protein, expressed_[ARAB]double-stranded-RNA-binding protein 4
2_51310	3_075370	PF03195 - protein of unknown function DUF260 (DUF260) (1 of 28)_[RICE]DUF260 domain containing protein, putative, expressed_[ARAB]LOB domain-containing protein 20
2_51320	3_075380	histone-lysine N-methyltransferase activity (Blast2GO)_[RICE]histone-lysine N-methyltransferase SUVR3, putative, expressed_[ARAB]SET domain protein 20
2_51331	–	PTHR11260//PTHR11260:SF279 - GLUTATHIONE S-TRANSFERASE, GST, SUPERFAMILY, GST DOMAIN CONTAINING//SUBFAMILY NOT NAMED (1 of 10)_[RICE]DNA binding protein, putative, expressed_[ARAB]DNA binding
2_51340	3_075330	ATP binding (Blast2GO)_[RICE]transcription initiation factor IIF beta subunit, putative, expressed_[ARAB]transcription initiation factor IIF, beta subunit
2_51350	3_075470/75500/75510	K18588 - coenzyme Q-binding protein COQ10 (COQ10) (1 of 1)_[RICE]cyclase/dehydrase family protein, expressed_[ARAB]polyketide cyclase /dehydrase and lipid transport protein
2_51360	3_075480	putative glycosyltransferase CAZy family GT14_[RICE]xylosyltransferase, putative, expressed_[ARAB]Core-2/I-branching beta-1,6-N-acetylglucosaminyltransferase family protein
**2_51370**	**3_075540**	**CK1_CaseinKinase_1a.7 - CK1 includes the casein kinase 1 kinases, expressed, subfamily CaseinKinase_1a_[RICE]CK1_CaseinKinase_1a.3 - CK1 includes the casein kinase 1 kinases, expressed_[ARAB]protein kinase family protein**
2_51377	–	PTHR10663:SF114 - PROTEIN MON2 HOMOLOG (1 of 1)_[RICE]guanine nucleotide exchange family protein, putative, expressed_[ARAB]ARM repeat superfamily protein
2_51390	3_075580	single-stranded DNA binding (Blast2GO)_[RICE]single-stranded DNA-binding protein, putative, expressed_[ARAB]mitochondrially targeted single-stranded DNA binding protein
2_51400	3_075590	GTPase activity (Blast2GO)_[RICE]signal recognition particle 54 kDa protein, putative, expressed_[ARAB]Signal recognition particle, SRP54 subunit protein
2_51410	3_075690	homocysteine S-methyltransferase activity (Blast2GO)_[RICE]homocysteine S-methyltransferase protein, putative, expressed_[ARAB]homocysteine S-methyltransferase 3
2_51417	3_075620	5.4.99.27 - tRNA pseudouridine(13) synthase /tRNA Psi(13) synthase (1 of 2)_[RICE]pseudouridylate synthase, putative, expressed_[ARAB]pseudouridine synthase family protein
2_51430	3_075710	PTHR12895:SF9 - DYMECLIN (1 of 1)_[RICE]dymeclin, putative, expressed_[ARAB]
2_51440	–	serine-type endopeptidase activity (Blast2GO)_[RICE]OsSub24 - putative subtilisin homologue, expressed_[ARAB]subtilase family protein
2_51450	3_075750	K18066 - tRNA wybutosine-synthesizing protein 5 [EC:1.14.11.42] (TYW5) (1 of 1)_[RICE]transcription factor jumonji, putative, expressed_[ARAB]2-oxoglutarate (2OG) and Fe(II)-dependent oxygenase superfamily protein
2_51467	3_075850	K10085 - ER degradation enhancer, mannosidase alpha-like 2 (EDEM2) (1 of 1)_[RICE]glycosyl hydrolase family 47 domain contain protein, expressed_[ARAB]Glycosyl hydrolase family 47 protein
2_51480	3_075870	K02721 - photosystem II PsbW protein (psbW) (1 of 2)_[RICE]photosystem II reaction center W protein, chloroplast precursor, putative, expressed_[ARAB]photosystem II reaction center W
2_51490	3_075840	2.7.1.176 - UDP-N-acetylglucosamine kinase /UNAG kinase (1 of 1)_[RICE]expressed protein_[ARAB]P-loop containing nucleoside triphosphate hydrolases superfamily protein
2_51500	3_075820/75830	PF03126 - plus-3 domain (Plus-3) (1 of 8)_[RICE]plus-3 domain containing protein, expressed_[ARAB]zinc knuckle (CCHC-type) family protein
2_51510	3_075790	protein kinase activity (Blast2GO)_[RICE]expressed protein_[ARAB]protein of unknown function, DUF593
2_51517	3_075800	KOG3740 - uncharacterized conserved protein (1 of 1)_[RICE]MATH domain containing protein, expressed_[ARAB]TRAF-like superfamily protein
2_51530	3_075920	delta24-sterol reductase activity, FAD binding (Blast2GO)_[RICE]cytokinin dehydrogenase precursor, putative, expressed_[ARAB]cytokinin oxidase 5
2_51540	3_075960	IgA binding (Blast2GO)_[RICE]jacalin-like lectin domain containing protein, expressed_[ARAB]mannose-binding lectin superfamily protein
2_51550	3_075910	Ran GTPase binding (Blast2GO)_[RICE]AGAP000951-PA, putative, expressed_[ARAB]LisH and RanBPM domains containing protein
**Marker = ctg50617 Trait = heading date (Y1) *P* = 2.41 × 10** ^**−5**^ **Physical ctg = 63852c-27**		
1_58190	2_060770	PTHR10994:SF62 - RETICULON-LIKE PROTEIN B8 (1 of 2)_[RICE]reticulon domain containing protein, putative, expressed_[ARAB]reticulon family protein
1_58197	–	PTHR13068//PTHR13068:SF37 - CGI-12 PROTEIN-RELATED//SUBFAMILY NOT NAMED (1 of 13)_[RICE]mTERF family protein, expressed_[ARAB]mitochondrial transcription termination factor family protein
1_58210	2_060710/60730	aldo-keto reductase activity (Blast2GO)_[RICE]oxidoreductase, aldo/keto reductase family protein, putative, expressed_[ARAB]NAD(P)-linked oxidoreductase superfamily protein
1_58220	2_060710/60730	aldo-keto reductase activity (Blast2GO)_[RICE]oxidoreductase, aldo/keto reductase family protein, putative, expressed_[ARAB]NAD(P)-linked oxidoreductase superfamily protein
**1_58230**	**2_060680**	**aka phytochrome interacting factor like_[RICE]helix-loop-helix DNA-binding domain containing protein, expressed_[ARAB]phytochrome interacting factor 3**
1_58240	2_060630/60650	–
1_58250	2_060620/60640	ATP-dependent RNA helicase activity (Blast2GO)_[RICE]DEAD-box ATP-dependent RNA helicase, putative, expressed_[ARAB]putative mitochondrial RNA helicase 2
1_58251	–	–
1_58252	–	PF07762 - protein of unknown function (DUF1618) (DUF1618) (1 of 77)_[RICE]expressed protein_[ARAB]
1_58254	–	PF07762 - protein of unknown function (DUF1618) (DUF1618) (1 of 77)_[RICE]expressed protein_[ARAB]
**Marker = ctg35543 Trait = WSC *P* = 2.66 × 10** ^**−5**^ **Physical ctg = 4029c-18**		
4_31640	5_066810/66820	microtubule minus-end binding (Blast2GO)_[RICE]Spc97/Spc98 family protein, putative, expressed_[ARAB]spindle pole body component 98
4_31645	5_066850	PTHR22763//PTHR22763:SF41 - RING ZINC FINGER PROTEIN//SUBFAMILY NOT NAMED (1 of 2)_[RICE]cytokinesis negative regulator RCP1, putative, expressed_[ARAB]RING-H2 finger C1A
4_31650	–	–
4_31660	5_066860/66920/66950	–
4_31670	5_066860/66920/66950	–
**4_31680**	**–**	**methenyltetrahydrofolate cyclohydrolase activity, ATP binding, methylenetetrahydrofolate dehydrogenase (NADP+) activity (Blast2GO)_[RICE]formate--tetrahydrofolate ligase, putative, expressed_[ARAB]10-formyltetrahydrofolate synthetase**
4_31690	5_067010	homeodomain-leucine zipper II family protein, subfamily A_[RICE]homeobox associated leucine zipper, putative, expressed_[ARAB]homeobox from Arabidopsis thaliana
4_31701	–	PF08879 - WRC (WRC) (1 of 21)_[RICE]_[ARAB]
4_31710	1_068030	steroid 17-alpha-monooxygenase activity (Blast2GO)_[RICE]cytochrome P450, putative, expressed_[ARAB]cytochrome P450, family 76, subfamily C, polypeptide 4
4_31720	1_068030	steroid 17-alpha-monooxygenase activity (Blast2GO)_[RICE]cytochrome P450, putative, expressed_[ARAB]cytochrome P450, family 76, subfamily C, polypeptide 4
**Marker = ctg8394 Trait = plant width *P* = 1.66 × 10** ^**−5**^ **Physical ctg = 2309c-18**		
1_34850	7_095230	PF02458 - transferase family (transferase) (1 of 99)_[RICE]transferase family protein, putative, expressed_[ARAB]HXXXD-type acyl-transferase family protein
1_34857	7_095190	PF01535//PF12854//PF13041//PF13812 - PPR repeat (PPR)//PPR repeat (PPR_1)//PPR repeat family (PPR_2)//pentatricopeptide repeat domain (PPR_3) (1 of 8)_[RICE]pentatricopeptide, putative, expressed_[ARAB]pentatricopeptide repeat (PPR-like) superfamily protein
1_34867	7_095200	–
1_34878	–	–
1_34890	7_095220	–
1_31050	–	PTHR20855//PTHR20855:SF55 - ADIPOR/PROGESTIN RECEPTOR-RELATED//SUBFAMILY NOT NAMED (1 of 1)_[RICE]haemolysin-III, putative, expressed_[ARAB]heptahelical protein 3
1_34900	7_095170/95180	K12868 - pre-mRNA-splicing factor SYF2 (SYF2) (1 of 2)_[RICE]pre-mRNA-splicing factor syf2, putative, expressed_[ARAB]GCIP-interacting family protein
1_30026	7_119150	PTHR22835//PTHR22835:SF116 - ZINC FINGER FYVE DOMAIN CONTAINING PROTEIN//SUBFAMILY NOT NAMED (1 of 3)_[RICE]GDSL-like lipase/acylhydrolase, putative, expressed_[ARAB]GDSL-like lipase/acylhydrolase superfamily protein
**Marker = ctg9479 Trait = heading date (Y2) *P* = 4.17 × 10** ^**−5**^ **Physical ctg = 2356c-18**		
4_26270	4_026720	glycosyl hydrolase (GH), subfamily GH51_[RICE]alpha-N-arabinofuranosidase A, putative, expressed_[ARAB]alpha-L-arabinofuranosidase 1
4_26275	–	PF02365 - no apical meristem (NAM) protein (NAM) (1 of 133)_[RICE]no apical meristem protein, putative, expressed_[ARAB]NAC domain containing protein 52
4_26280	–	pectin methylesterase inhibitor (PMEI)_[RICE]PME/invertase inhibitor, putative, expressed_[ARAB]
4_26287	4_026690	PF05701 - weak chloroplast movement under blue light (WEMBL) (1 of 14)_[RICE]prefoldin, putative, expressed_[ARAB]Plant protein of unknown function (DUF827)
**4_26300**	**4_026680**	**PINFORMED-like auxin efflux carrier, syntenic to Sb05g002150, Os11g04190, Os12g04000_[RICE]auxin efflux carrier component, putative, expressed_[ARAB]auxin efflux carrier family protein**
4_26310	4_026630	–
4_26317	4_026640	PTHR24349//PTHR24349:SF130 - SERINE/THREONINE-PROTEIN KINASE//SUBFAMILY NOT NAMED (1 of 2)_[RICE]CAMK_CAMK_like.42 - CAMK includes calcium/calmodulin dependent protein kinases, expressed_[ARAB]calcium-dependent protein kinase 17
4_26330	4_026660	holo-[acyl-carrier-protein] synthase activity (Blast2GO)_[RICE]dehydrogenase-phosphopantetheinyltransferase, putative, expressed_[ARAB]4\’-phosphopantetheinyl transferase superfamily
4_26342	4_026570/26340/26390	KOG2615 - permease of the major facilitator superfamily (1 of 10)_[RICE]major facilitator superfamily antiporter, putative, expressed_[ARAB]zinc induced facilitator-like 1
4_26354	4_026570/26340/26390	KOG2615 - permease of the major facilitator superfamily (1 of 10)_[RICE]major facilitator superfamily antiporter, putative, expressed_[ARAB]zinc induced facilitator-like 1
4_26366	4_026570/26390/26340	KOG2615 - permease of the major facilitator superfamily (1 of 10)_[RICE]major facilitator superfamily antiporter, putative, expressed_[ARAB]zinc induced facilitator-like 1
4_26369	4_026340	–
4_26380	4_026390/26340	tetracycline:hydrogen antiporter activity (Blast2GO)_[RICE]major facilitator superfamily antiporter, putative, expressed_[ARAB]zinc induced facilitator-like 1
4_26390	4_026330	phosphoinositide 3-kinase binding, actin filament binding (Blast2GO)_[RICE]WD domain, G-beta repeat domain containing protein, expressed_[ARAB]transducin family protein/WD-40 repeat family protein
4_26400	4_026290	myosin heavy chain binding, actin filament binding (Blast2GO)_[RICE]OsCML2 - calmodulin-related calcium sensor protein, expressed_[ARAB]calmodulin 5
4_26410	4_026300	CAMK_KIN1/SNF1/Nim1_like.32 - CAMK includes calcium/calmodulin dependent protein kinases, expressed, subfamily KIN1/SNF1/Nim1_like(CAMK_2)_[RICE]CAMK_KIN1/SNF1/Nim1_like.37 - CAMK includes calcium/calmodulin dependent protein kinases, expressed_[ARAB]CBL-interacting protein kinase 3
**Marker = ctg54379 Trait = biomass *P* = 3.71 × 10** ^**−5**^ **Physical ctg = 896c-18**		
2_50230	3_072780	–
2_50237	3_072800	PTHR22792:SF58 - LA-RELATED PROTEIN 6A (1 of 1)_[RICE]la domain containing protein, expressed_[ARAB]RNA-binding protein
2_50250	3_072860	PF04862 - protein of unknown function (DUF642) (DUF642) (1 of 9)_[RICE]expressed protein_[ARAB]protein of unknown function, DUF642
2_50260	3_072850	intracellular cyclic nucleotide activated cation channel activity, voltage-gated potassium channel activity (Blast2GO)_[RICE]potassium channel KAT1, putative, expressed_[ARAB]potassium channel in *Arabidopsis thaliana* 1
2_50270	–	–
**2_50280**	**3_072810**	**PTHR10209:SF171 - GIBBERELLIN 2-BETA-DIOXYGENASE 2-RELATED (1 of 1)_[RICE]gibberellin 2-beta-dioxygenase, putative, expressed_[ARAB]gibberellin 2-oxidase**
2_50290	3_072910	microtubule-severing ATPase activity (Blast2GO)_[RICE]AAA-type ATPase family protein, putative, expressed_[ARAB]P-loop containing nucleoside triphosphate hydrolases superfamily protein
2_50300	3_072900	K04507 - calcyclin binding protein (CACYBP, SIP) (1 of 1)_[RICE]SGS domain containing protein, expressed_[ARAB]SGS domain-containing protein
2_50304	–	–
2_50308	–	–
2_50317	3_072880	–
2_50330	3_072930	PF01535//PF13812 - PPR repeat (PPR)//pentatricopeptide repeat domain (PPR_3) (1 of 23)_[RICE]PPR repeat domain containing protein, putative, expressed_[ARAB]pentatricopeptide repeat (PPR) superfamily protein
2_50340	3_072920	DNA-directed DNA polymerase activity (Blast2GO)_[RICE]DNA-directed polymerase, putative, expressed_[ARAB]Y-family DNA polymerase H
2_35187	1_022100	K16571 - gamma-tubulin complex component 4 (TUBGCP4, GCP4) (1 of 1)_[RICE]Spc97/Spc98 family protein, putative, expressed_[ARAB]GAMMA-TUBULIN COMPLEX PROTEIN 4
**Marker = ctg20671 Trait = heading date (Y2) *P* = 5.15 × 10** ^**−5**^ **Physical ctg = 1969c-18**		
1_14590	–	N-acetyl-gamma-glutamyl-phosphate reductase activity (Blast2GO)_[RICE]semialdehyde dehydrogenase, NAD binding domain containing protein, putative, expressed_[ARAB]oxidoreductases, acting on the aldehyde or oxo group of donors, NAD or NADP as acceptor;copper ion binding
3_15607	7_116040	6.3.2.5 - phosphopantothenate–cysteine ligase/phosphopantothenoylcysteine synthetase (1 of 2)_[RICE]phosphopantothenate–cysteine ligase, putative, expressed_[ARAB]4-phospho-panto-thenoylcysteine synthetase
3_15620	2_116060	DNA-directed DNA polymerase activity, 3′-5′ exonuclease activity (Blast2GO)_[RICE]PolI-like DNA polymerase, putative, expressed_[ARAB]polymerase gamma 2

^1^Gene model identifiers are abbreviated as C_nnnnn(n), where C is the chromosome and nnnnn(n) is the identifier number. Gene models highlighted in bold are discussed as candidate genes.

^2^Functional annotations obtained from Bdistachyon_314_v3.1.

BLAST searches of LpBAC5000 against the published *L. perenne* draft genome sequence were also used to suggest scaffolds from this draft genome assembly that were contiguous according to the physical map contigs. The numbers of draft genome scaffolds aligned varied from nine for 4073c-18 to one for 1969c-18 (Fig. 3, Supplementary Data Fig. S3).

## DISCUSSION

The aim of this paper has been to explore the targeted use of *L. perenne* BAC-based physical maps to suggest broader genomic regions, validated through the physical contig structures, that could be associated with markers found to be significant in a GWAS analysis derived from the field performance of a selection of European *L. perenne* ecotypes. To this end, the physical map assemblies and associated sequence tags presented in the paper encompass the majority of the *L. perenne* genome and extend the ‘genomic reach’ of the current published sequence-based assemblies for *L. perenne* (Byrne *et al.*, 2015; Velmurugan *et al.*, 2016). As a result, we have been able to identify both genomic regions and relevant gene models contained within these regions, which are associated with the GWAS markers and phenotypes. As a significance level we chose to use a 10 % FDR as, in taking this work forward, this represents a good balance between the likelihood of identifying useful variation and the resources required for screening plant material in follow-up evaluations. In terms of candidate genes, as always for non-model species (and particularly so for non-cereal grass species) there is a frustrating gap between our ability to identify candidate genes and our subsequent ability to validate these candidate genes experimentally. However, even in the absence of direct functional validation of candidates, knowledge of the regions of genomes that contribute to trait performances are valuable in identifying potentially useful genotypes/phenotypes present in germplasm collections for further experimentation.

Blackmore *et al.* (2015, 2016) reported the establishment of this panel of 716 *L. perenne* ecotypes sampled from 90 locations across Europe and described the genetic relationships between population and patterns of linkage disequilibrium on the basis of the genotype scores from the Infinium marker array. The present study, in part, represents a follow-up analysis incorporating marker and phenotype data, but also integrates this within a genomic context. As is common to a number of population genetics analyses within the ryegrasses, flowering time features strongly in terms of traits that can be associated with genetic markers and genomic regions, and four of the eight loci within the 10 % FDR were associated with heading date, one from year 1 and three from year 2. As an outcome this is not particularly surprising, as the phenology of flowering is known to be a key determinant of many growth, physiological and biochemical parameters in the annual cycle of perennial grasses. Commercially, the selection of genotypes that have co-ordinated flowering times represents a primary route for controlling phenotypic variation. In addition to flowering time, significant marker/trait associations were identified for plant width, plant biomass and WSC content. The integration of the physical mapping with the GWAS is discussed below on a marker-by-marker/contig-by-contig basis.

### Marker ctg8613; physical contig 4073c-18; trait, heading date year 2 (Fig. 3A)

Marker ctg8613 had its closest BLAST alignment with gene model Bradi2g51467, which associated it with physical contig 4073c-18. This was a relatively large physical contig consisting of 138 BACs and spanning 2227 high-information-content (HIC) fingerprints. In terms of conserved synteny with *B. distachyon*, this contig spanned a region containing 36 gene models from Bradi2g51255 to Bradi2g51550, 32 of which were tagged within LpBAC5000 (Table 4). Twenty-seven of these gene models were associated with annotations, one of which (Bradi2g51370/HORVU3Hr1G075540) represents a member of the *Casein Kinase 1* gene family (*CK1*). Members of this gene family have been directly associated with the modulation of flowering time in both *Arabidopsis thaliana* (hereafter referred to as arabidopsis) and rice (Tan *et al.*, 2013; Kwon *et al.*, 2015; Zhang *et al.*, 2016) and, in the latter case, a *CK1* gene underlies the Hd16/Early Flowering 1 heading date QTL (Dai and Xue, 2010; Hori *et al.*, 2013). Marker ctg8613 has not previously been genetically mapped in *L. perenne* but superpool/matrix pool screens of a different marker, R1_302, identified three independent BACs from contig 4073c-18. This would place this contig on chromosome 3 of the *Lolium/Festuca* introgression map (King *et al.*, 2013). Additionally, marker ctg41342 could also be aligned with physical contig 4073c-18 and this has been previously mapped in the AberMagic × Aurora genetic mapping population to chromosome 3 at 49 cM (marker ctg41342 was 79 % homozygous for one of the alleles in the present study and was not significantly associated with heading date). This placing of physical contig 4073c-18 on chromosome 3 would not suggest that this *CK1* is a direct orthologue of the gene underlying the Hd16 QTL in rice (Os03g0793500/LOC_Os03g57940), but is consistent with orthology with Os01g0772600/LOC_Os01g56580. While we are not aware of any rice heading date QTL directly associated with Os01g0772600/LOC_Os01g56580, it is the most closely related gene model to Hd16, showing 77 % sequence identity at the amino acid level. Thus, a functional role in flowering regulation is not unlikely. Additionally, the closest orthologue in arabidopsis to Bradi2g51370/HORVU3Hr1G075540/Os01g0772600 is AT3G03940. This arabidopsis gene model is annotated as a protein kinase family protein, specifically as photoregulatory protein kinase 3 (PPK3). The PPK protein family has been shown in arabidopsis to interact in the blue-light-dependent phosphorylation of cryptochrome 2, a protein involved in photoperiod-induced flowering, and *ppk* mutants are associated with delayed flowering (Tan *et al.*, 2013). Thus, the *CK1* gene model identified on physical contig 4073c-18 is a good candidate for influencing flowering time in *L. perenne*.

### Marker, ctg50617; physical contig, 63852c-27; trait, heading date year 1 (Supplementary Data Fig. S3A)

A second physical contig associated with heading date consisted of 65 BACs spanning 980 HIC fingerprints that showed conserved synteny with a region of the *B. distachyon* genome from Bradi1g58190 to Bradi1g58254, including ten gene models, eight of which were tagged in LpBAC5000. Of the eight gene models in this region with annotations, one, Bradi1g58230/HORVU2Hr1G060680, is predicted to code for a phytochrome-interacting factor (PIF). The PIFs are a family of transcription factors that interact with the red/far-red light receptors, phytochromes, and so are involved in light perception and photomorphogenesis (Pham *et al.*, 2018). In arabidopsis, PIF3 has been associated particularly with seedling de-etiolation, chlorophyll and anthocyanin biosynthesis and, through interaction with circadian clock genes, diurnal physiological responses (Soy *et al.*, 2016). It has also been shown in arabidopsis that both constitutive and tissue-specific expression of *PIF3* in the plant vasculature can promote flowering; *pif3* mutants can also show delayed flowering. In rice, the putative *PIF3* orthologue Os07g0143200/LOC_Os07g05010, known as *OsPIF14* (Cordeiro *et al.*, 2016) or *Phytochrome Interacting Factor 3-Like 14* (*OsPIL14*; Nakamura *et al.*, 2007), has been suggested as being involved in the interaction between light and stress signalling through repression of *Dehydration-Responsive Element-Binding 1/C-Repeat-Binding Factor* (*DREB1/CBF*) genes (Cordeiro *et al.*, 2016). This represents a possible route through which the *PIF3* gene model within this region in *L. perenne* could have a significant effect on flowering time in this ecotype population.

### Marker, ctg9479; physical contig, 2356c-18; trait, heading date year 2 (Fig. 3B)

A third physical contig associated with heading date consisted of 54 BACs spanning 977 HIC fingerprints and showed conserved synteny with a region of the *B. distachyon* genome from Bradi4g26270 to Bradi4g26410. The region contained 16 gene models, 13 of which were tagged within LpBAC5000. Among these, gene model Bradi4g26300/HORVU4Hr1G026680 is annotated as a PIN-FORMED auxin efflux carrier protein. Polar auxin transport is established as being a key process in plant morphogenesis in which differential auxin gradients determine the developmental differentiation of meristems (Balzan *et al.*, 2014). Bradi4g26300 itself belongs to the Sister-of-PIN1 (SoPIN1) clade and *sopin1* mutants in *B. distachyon* have been associated with abnormal spikelet development (O’Connor, 2014), and it is not unlikely that the orthologue of this gene in *L. perenne* is also involved in determining floral meristem development. Expressions of *PIN* genes are known to be influenced by abiotic stresses, implying that there can be an environmentally induced response in terms of auxin gradients within plant tissues (Wang *et al.*, 2015). Particularly, for such a diverse pan-European ecotype collection grown in a single (UK) environment, a proportion of the genotypes are likely to have been under some degree of environmental stress simply due to the contrasts between the collection and evaluation sites. Thus, environmentally induced alterations in the expression patterns at the floral meristem of the *L. perenne* orthologue of Bradi4g26300 could result in changes in flowering patterns.

### Marker ctg35543; physical contig 4029c-18; trait, WSC (Supplementary Data Fig. S3B)

This physical contig consisted of 58 BACs spanning 978 HIC fingerprints. In terms of conserved synteny with *B. distachyon*, 4029c-18 included gene models from Bradi4g31640 to Bradi4g31720. Only six of the ten gene models within this region were tagged within LpBAC5000, partly due to lack of MTP coverage at one end of this contig. However, one of the BES within this region could be aligned with Bradi4g31720 (and Scaffold_3258_ref0017503, from Byrne *et al.*, 2015), thus establishing the region of conserved synteny at the end of the contig that lacked MTP coverage. The most interesting annotated gene model within this region was Bradi4g31680, to which the most similar gene model in arabidopsis is AT1G50480, itself annotated as 10-formyltetrahydrofolate synthetase (THFS). THFS catalyses the conversion of formate and tetrahydrofolate to 10-formyl tetrahydrofolate and is an important enzyme in one-carbon metabolism (Hanson and Roje, 2001). The reverse of this reaction is catalysed by 10-formyl tetrahydrofolate deformylases, which have been established as being essential enzymes in photorespiration in arabidopsis under ambient CO_2_ concentration. Knockouts of 10-formyl tetrahydrofolate deformylases result in accumulation of glycine and 5- and 10-formyl tetrahydrofolate (Collakova *et al.*, 2008). While THFS is directly metabolically linked through to methionine/serine/glycine/purine metabolism, in general terms it is an enzyme that affects the partitioning of carbon between different pools in plants. Additionally, it could also influence photosynthetic efficiency through metabolic feedback on photorespiration. Thus, variation in accumulation of WSCs across this ecotype collection could be a function of overall photosynthetic efficiency and assimilate partitioning influenced by differential THFS activity. Interestingly, Blackmore *et al.* (2015) identified marker ctg35543 as being one of the top 50 markers contributing to the first principal component (associated with east–west geographical distribution) in their analysis of genetic diversity. This is a further indication of the potential significance of this gene.

### Marker ctg54379; physical contig 896c-18; trait, biomass (Supplementary Data Fig. S3C)

Physical contig 896c-18 consisted of 60 BACs and spanned 1180 HIC fingerprints. It contained a conserved syntenic region covering Bradi2g50230 to Bradi2g50340 but also included a not-immediately-co-linear gene model at the end of the contig, Bradi2g35187. The region contained 14 gene models, 11 of which were tagged within LpBAC5000. Among the gene models, Bradi2g50280 was annotated as a gibberellin 2-oxidase (GA2ox). The closest orthologue in rice to Bradi2g50280 is LOC_Os01g55240/Os01g0757200, which is identified as gibberellin 2-oxidase 3 (GA2ox3; Lo *et al.*, 2008). The GA2ox family in rice is responsible for regulating the levels of biologically active gibberellins in tissues by metabolizing active gibberellin (GA) forms to inactive ones. Specifically, GA2ox3 is an important enzyme in the conversion of biologically active GA1 and GA4 to the inactive GA34 and GA8 (Lo *et al.*, 2008). In general, GA deficiency in rice is associated with dwarf phenotypes, but there can also be effects on seed dormancy and shoot and root growth, including tillering, flowering and seed-set. Specifically, a *GA2ox3* T-DNA insertion mutant in rice was found to have a severe dwarf phenotype with no seed production (Lo *et al.*, 2008) and a rice GWAS study identified SNP markers close to *GA2ox3* that were significantly associated with seed dormancy (Magwa *et al.*, 2016). Marker ctg54379 was significantly associated with vegetative biomass measured in June of year 1. Biomass in ryegrass is a function of overall leaf growth, but also of tiller number. The GA2ox family will play an important role in the regulation of growth and architectural parameters in ryegrasses through their role in gibberellin metabolism, as they do in other plant species. However, the QQ plot for this trait (Fig. 2) does not obviously show the ‘elbow’ shape associated with the presence of unusually significant markers. Therefore, while there is close proximity between the *GA2ox3* gene and the significant marker for biomass, the interpretation of this marker/trait association has to be treated with caution.

### Marker ctg8394 and marker ctg20671

Marker ctg8394 and marker ctg20671 were associated with physical contigs 2309c-18 (Supplementary Data Fig. S3D) and 1969c-18 (Supplementary Data Fig. S3E), respectively. Both physical contigs had some inconsistencies in terms of conserved synteny with *B. distachyon*, and 2309c-18 showed inconsistent clone order and composition across the various physical map assembly parameters (Supplementary Data Table S4). Physical contig 1969c-18 was, seemingly, a reliable contig; however, gene models could only be tagged within LpBAC5000 at one end of the contig. None of the gene model annotations could be obviously related to possible functions in moderating the associated traits for either physical contig. These contigs are not discussed further.

### QTL and GWAS studies in *L. perenne*

#### Heading date.

There have been a considerable number of published studies of the genetic control of flowering in *L. perenne* and related grasses, mostly based upon QTL analyses in bi-parental genetic mapping families (see Shinozuka *et al.*, 2012, for a comprehensive overview). Heading date QTL have been identified on all seven *L. perenne* linkage groups (LGs) and, in the present study, significant markers were associated with LGs 2, 3, 4 and 7. Often, the lack of common markers across studies and the low resolution of many QTL analyses presents problems in terms of identifying equivalent QTL, and this is certainly true when trying to put the results of the present study in a broader context. We can say that the assumed genetic position of marker ctg8613-723 on LG3 is not incompatible with the position of the heading-date QTL identified on the same LG associated with marker LpRGA5 in the study of Studer *et al.* (2008). Similarly, the genetic position of ctg20671-156 on LG7 is not incompatible with the minor heading-date QTL identified on LG7 associated with markers LTCOa/b in the study of Armstead *et al.* (2004). More certainly, we can say that none of the significant heading-date markers from the present study can be associated, in terms of genetic/genomic position, with the major heading-date QTL that have been identified on LG4 and LG7, for which there are good candidate genes, i.e. the *L. perenne* orthologues of *Vernalisation 1* and *Flowering Locus T*, respectively (Armstead *et al.*, 2004; Jensen *et al.*, 2005; Skøt *et al.*, 2011). More recent studies have analysed GWAS data for significant associations with flowering time. Arojju *et al.* (2016) used six full-sib families derived from crosses between perennial ryegrass varieties and did not identify any significant marker/trait associations in the GWAS. However, using a single-marker analysis approach, they did identify significant associations on LG2, 4 and 7. Similarly, Fè *et al.* (2015) presented a GWAS study using a mixture of *F*_2_ families and synthetic populations, both derived from breeding material, and identified 19 SNPs significantly (5 % FDR) associated with heading date. In the study of Fè *et al.* (2015) the relationship of these SNPs to the published *L. perenne* sequence scaffolds (Byrne *et al.*, 2015) was provided. However, none of these scaffolds could be associated with the physical contigs identified in the present study.

#### Plant width, biomass and WSC.

Marker ctg8394-538 was associated with plant width, which is a function of both tiller number and growth habit (i.e. prostrate versus erect). QTL for tiller number have been identified in different studies on all LGs of *L. perenne* and *Lolium multiflorum* (Inoue *et al.*, 2004; Yamada *et al.*, 2004; Kobayashi *et al.*, 2011; Sartie *et al.*, 2011), but the lack of common markers means that direct comparisons with the present study have very little resolution. However, in addition to tiller number, Yamada *et al.* (2004) also recorded prostrate versus erect growth habit and identified QTL for this trait on LG4 and 7. The QTL on LG7 had the highest LOD score of any QTL in that study and was also the only QTL that was non-co-incident with any of the other morphological traits measured. The position of this QTL [derived from one RFLP marker, RZ144, that can be linked through from the study of Yamada *et al.* (2004) to a previous version of the *F*_2_ genetic map] is not incompatible with the genetic position of marker ctg8394-538, though no obvious candidate gene was identified on the associated physical contig, 2309c-18.

Marker ctg54379-73 was significantly associated with plant biomass, though the QQ plot indicates that this may have been a chance outcome. Biomass is a trait that is a fundamental productivity measure for forage grasses and, not surprisingly, QTL have been identified on a number of different *L. perenne* LGs, including LG3. Two studies (Turner *et al.*, 2008; Anhalt *et al.*, 2009) associated the QTL peak for biomass with SSR marker rv1133. In a combined *F*_2_ genetic map that integrated some of the Infinium iSelect markers with previously genetically mapped markers (unpublished), rv1133 and ctg54379-73 genetically map ~20 cM apart. Therefore, the previously identified biomass QTL are probably not co-incident with any effect associated with marker ctg54379-73. Sartie *et al.* (2011) identified a QTL for tiller number (which is a component of biomass) on LG3, but there are no common markers that cross-reference to this study.

Ctg35543-1175 and ctg41386-226 identified significant associations with WSC on LG5 and LG6 respectively. Foito *et al.* (2015) and Turner *et al.* (2006) conducted a QTL analysis for WSC accumulation and polar metabolite concentrations (including soluble sugars) in *L. perenne* genetic mapping populations and identified QTL for various WSC fractions on LG1, 2, 3, 5, 6 and 7, which, specifically, included a QTL on LG5 for leaf glucose content in the autumn and QTL for total WSC, fructan, glucose and fructose on LG6. However, as for a number of the studies referred to in this discussion, lack of common markers makes establishing co-incidence of markers and QTL across studies problematic.

### Future directions

In this study we have described the development and deployment of a BAC-based physical mapping approach in order to inform and increase the resolution of a GWAS in terms of identifying potentially useful germplasm accessions, candidate genomic regions and genes. Clearly this is not an end-point for *L. perenne* genomics, the ultimate goal being to be able to define an accurate genomic sequence for *L. perenne* in the form of seven pseudomolecules and to integrate this with both comprehensive gene annotations and descriptions of physical genetic variation. Work is currently in progress to reach these ends at a number of research centres and it is hoped that the resources reported here will aid in the achievement of these aims. Beyond genomics, major challenges also exist for grassland geneticists and biotechnologists in understanding and exploiting the considerable germplasm resources that are available. These challenges include both the establishment of protocols and, hopefully, pipelines for higher-throughput phenotyping of *L. perenne* accessions and, at the molecular level, methods for validating candidate genes. In the latter context, while genetic transformation has been available for many years for *L. perenne* and related species and so the potential to knock out or otherwise modify the expression of specific genes is there, this is often quite a blunt tool, particularly if one is trying to understand the contribution of allelic differences to trait variation. For this, the application of Crispr Cas-9 and similar genome editing technologies (Yin *et al.*, 20 17) is likely to be the biotechnological route to a greater understanding. Thus, it is hoped that in the not too distant future we will be able to integrate phenotyping, genetics, genomics and biotechnology to more fully understand the biology of these grasses and so to contribute germplasm-based solutions to the various societal and environmental challenges and opportunities associated with grasslands.

## SUPPLEMENTARY DATA

Supplementary data are available online at https://academic.oup.com/aob and consist of the following. Fig. S1: distribution of assembly lengths and N50s for sequenced BACs in the MTP. Fig. S2: QQ plots for non-significant marker/trait associations from the GWAS. Fig. S3: diagrammatic representations of physical map ctgs 62852c-18, 4029c-18, 896c-18, 2309c-18 and 1969c-18 in relation to conserved syntenic regions in the *B. distachyon* genome. Methods S1: details of physical map construction using FPC and LTC. Methods S2: methods used for concatenation of BAC assemblies for comparison with the published assembly of Byrne *et al.* (2015) and for screening of MTP clones for potential cross-contamination. Methods S3: construction of BAC superpools and matrix pools for marker screening and details of the RAD sequencing pilot study. Methods S4 and S5: field and analytical chemistry measurements and protocols used in the evaluation of the ecotype family. Results S1: concatenation of BAC assemblies, identification and removal of potential cross-contaminating sequences and RAD sequencing pilot study. Table S1: map positions, marker sequences and SNP positions for markers assayed on the BAC superpools and matrix pools using KASP. Table S2: geographical collection sites of *L. perenne* accessions used in the GWAS analysis. Tables S3, S5 and S6: summary statistics for BAC library production and fingerprinting, sequence size range in the LpBAC5000 database and estimates of the *L. perenne* genome coverage within the physical maps. Supplementary Data available via doi consist of the following. Table S4: *L. perenne* physical maps generated using FPC and LTC assembly softwares from BAC HICF data, https://doi.org/10.20391/bb56e6d7-8913-4bd7-8167-2b7e4c01382b; LpBAC5000 sequence database, https://doi.org/10.20391/dfb05330-7485-444f-a475-8310bee5d510; BAC-end sequence database, https://doi.org/ 10.20391/61921116-ddd0-4d85-b0fd-e0d734bc63c8.

## Supplementary Material

mcy230_suppl_Supplementary_Figure_S1

mcy230_suppl_Supplementary_Figure_S2

mcy230_suppl_Supplementary_Figure_S3

mcy230_suppl_Supplementary_Methods_S1

mcy230_suppl_Supplementary_Methods_S2

mcy230_suppl_Supplementary_Methods_S3

mcy230_suppl_Supplementary_Methods_S4-S5

mcy230_suppl_Supplementary_Results_S1

mcy230_suppl_Supplementary_Table_S1

mcy230_suppl_Supplementary_Table_S2

mcy230_suppl_Supplementary_Table_S3
