# Supplementary material for: Integrating a newly developed BAC-based physical mapping resource for Lolium perenne with a genome-wide association study across a L. perenne European ecotype collection identifies genomic contexts associated with agriculturally important traits
Source: Ann Bot. 2019 Feb 2;123(6):977–92. doi: 10.1093/aob/mcy230 (PMC6589518; doi:10.1093/aob/mcy230)
Supplement: mcy230_suppl_Supplementary_Figure_S1 [file mcy230_suppl_supplementary_figure_s1.pptx]

## Slide 1
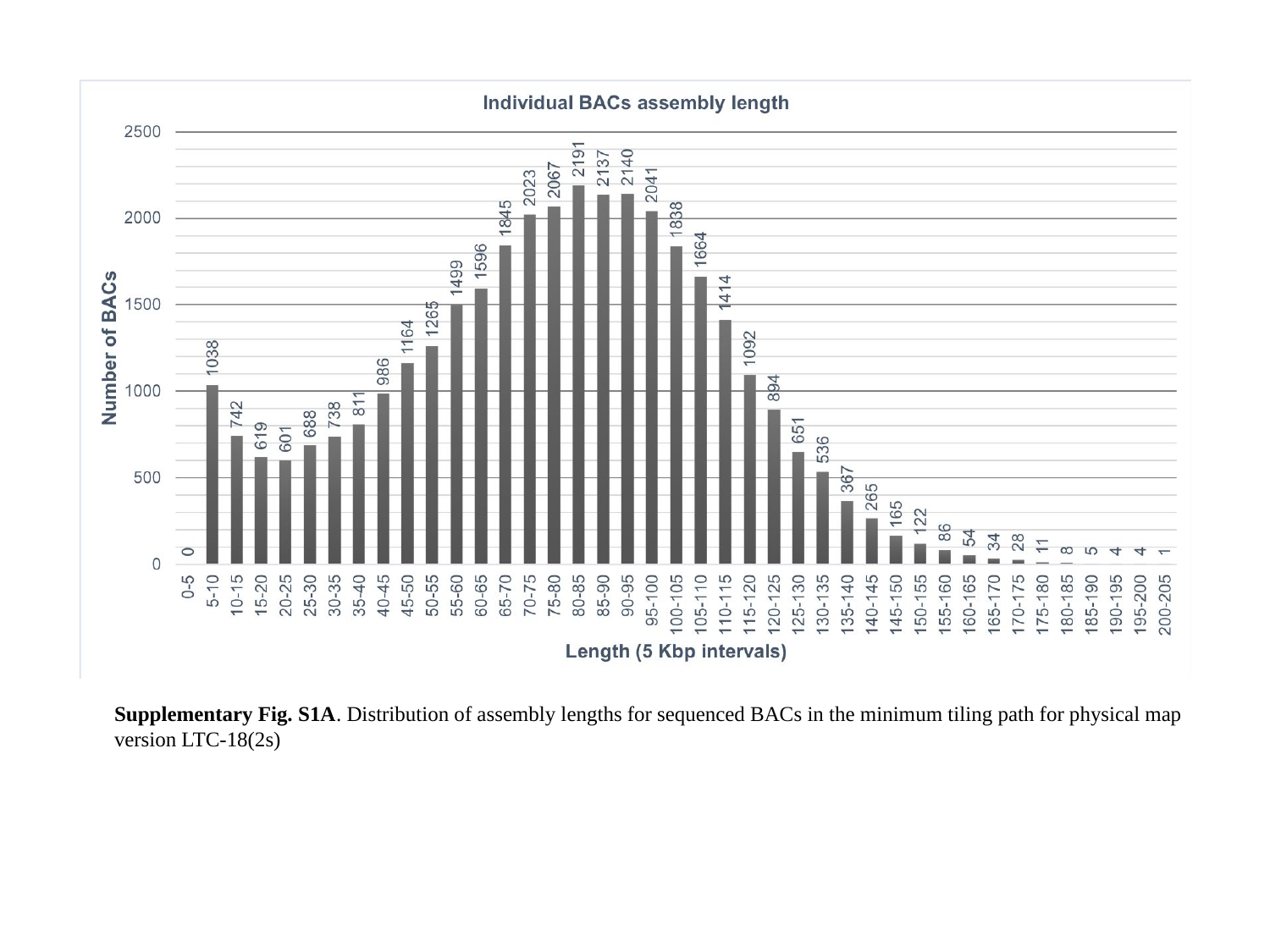

Supplementary Fig. S1A. Distribution of assembly lengths for sequenced BACs in the minimum tiling path for physical map version LTC-18(2s)

## Slide 2
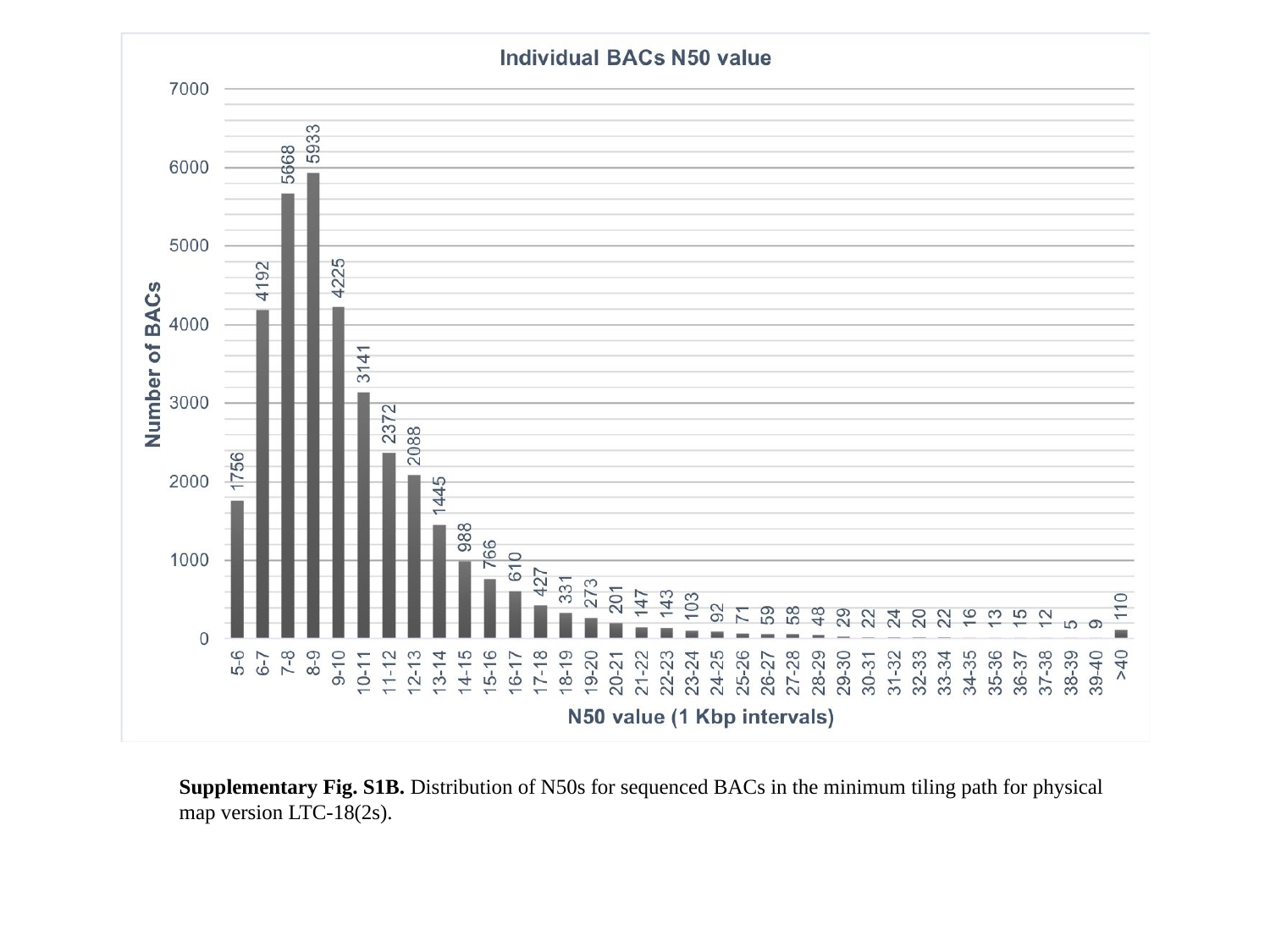

Supplementary Fig. S1B. Distribution of N50s for sequenced BACs in the minimum tiling path for physical map version LTC-18(2s).
