# Supplementary material for: Integrating a newly developed BAC-based physical mapping resource for Lolium perenne with a genome-wide association study across a L. perenne European ecotype collection identifies genomic contexts associated with agriculturally important traits
Source: Ann Bot. 2019 Feb 2;123(6):977–92. doi: 10.1093/aob/mcy230 (PMC6589518; doi:10.1093/aob/mcy230)
Supplement: mcy230_suppl_Supplementary_Figure_S3 [file mcy230_suppl_supplementary_figure_s3.docx]

***B. distachyon* genomic region**


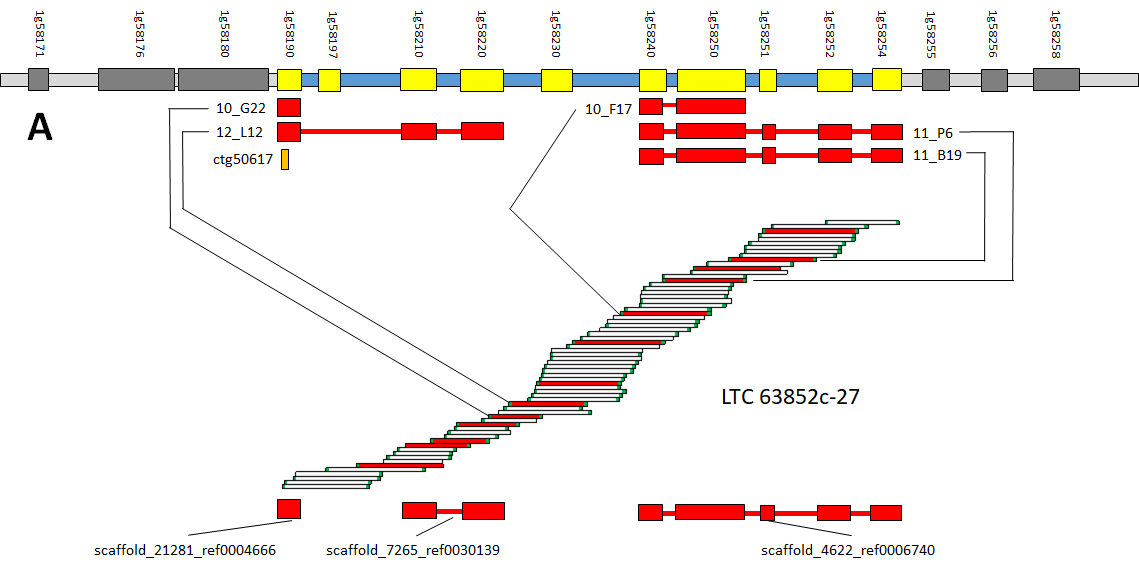


**LpBAC5000 alignments**

**Byrne et al. (2015) alignments**

***B. distachyon* genomic region**


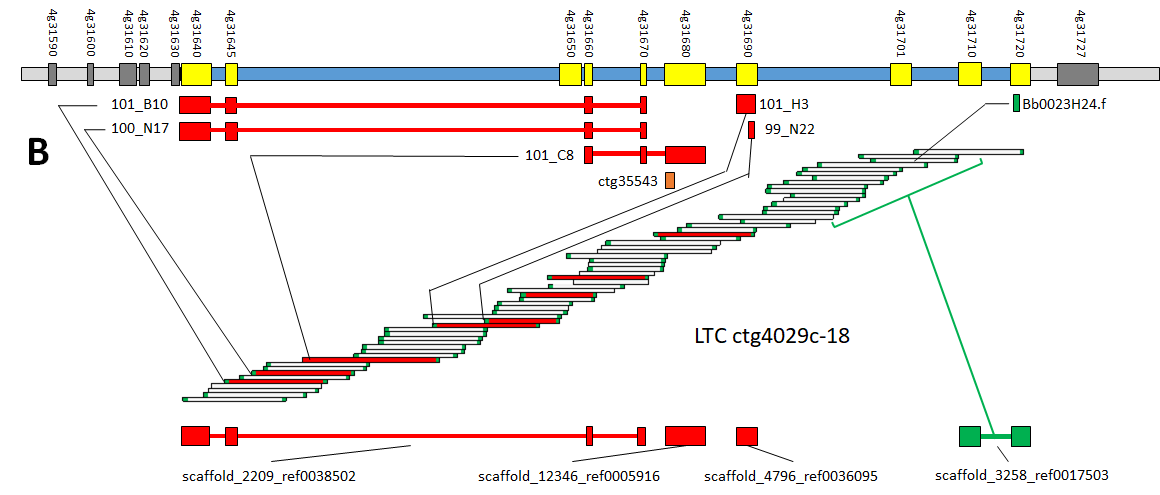


**BES**

**alignments**

**Byrne et al. (2015) alignments**

**LpBAC5000 alignments**


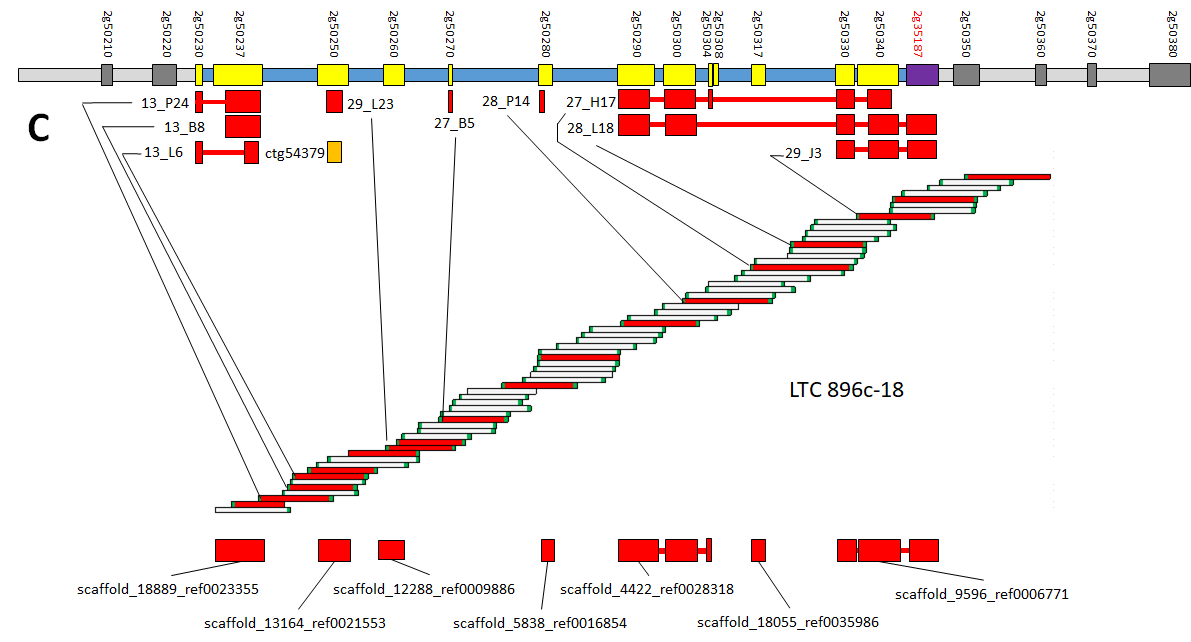


**Byrne et al. (2015) alignments**

**LpBAC5000 alignments**

***B. distachyon* genomic region**

***B. distachyon* genomic region**

**Byrne et al. (2015) alignments**

**LpBAC5000 alignments**

***B. distachyon* genomic region**

**Byrne et al. (2015) alignments**

**LpBAC5000 alignments**

**Supplementary Fig. S3.** Diagrammatic representations of physical map ctgs **A –** 62852c-18, **B** - 4029c-18, **C** – 896c-18, **D** – 2309c-18 and **E** – 1969c-18 in relation to conserved syntenic regions in the *B. distachyon* genome. Horizontal bars at the top of each figure represent the genomic region containing the *B. distachyon* gene models described. Yellow rectangles on the blue background illustrate the positions of the *B. distachyon* gene models within the defined region (the prefix ‘Bradi’ is omitted from all gene model identifiers for clarity). Dark grey rectangles on the light grey background illustrate the positions of *B. distachyon* gene models just outside of the defined region. Wide horizontal red bars illustrate the *B. distachyon* gene models tagged within LpBAC5000 or within the *L. perenne* genomic scaffolds from Byrne et al. (2015), above and below the contig illustration, respectively. Narrow horizontal red bars indicate where gene space sequences are contiguous. Orange filled boxes indicated the aligned position of the marker sequence. Red BACs within the contig illustrations indicate BACs for which sequence information is available. The BACs from which the aligned LpBAC5000 contigs were derived are given by the side of the aligned sequences. For **B**, green filled boxes indicate a region aligned through a BAC-end sequence. For **C** and **D,** the purple filled boxes relating to *B. distachyon* gene models indicate a break in the co-linearity.
