# Supplementary material for: Integrating a newly developed BAC-based physical mapping resource for Lolium perenne with a genome-wide association study across a L. perenne European ecotype collection identifies genomic contexts associated with agriculturally important traits
Source: Ann Bot. 2019 Feb 2;123(6):977–92. doi: 10.1093/aob/mcy230 (PMC6589518; doi:10.1093/aob/mcy230)
Supplement: mcy230_suppl_Supplementary_Methods_S1 [file mcy230_suppl_supplementary_methods_s1.docx]

**Supplementary Methods S1**

**Physical map construction using FPC**

FPC: FPC v 9.4 was obtained from <http://www.agcol.arizona.edu/software/fpc/>. An initial series of contigs was constructed using a p cut-off value of 1e^-50^ and the contigs were tested for the presence of ‘Q’ (query) clones using stepwise decreases in the p-value at 1x10^-53^, 1x10^-56^, 1x10^-59^ and identified Q clones were removed from the contigs (map version designated FPC1.01). Contigs were then subjected to the automerge procedure at the less stringent p-value of 1e^-23^, integration of singleton clones also at 1x10^-23^ (to derive map version FPC1.02) followed by a further round of automerging at 1x10^-18^ (map version FPC1.03).

**Physical map construction using LTC**

LTC: LTC software was obtained from MultiQTL Ltd, Haifa, Israel. Initial LTC map construction was performed using LTCbeta 2.1 and subsequent LTC maps were constructed using LTC 1.4.6.

For the initial LTC map construction using LTCbeta 2.1, it was found that the software could not process the complete database of clone band sizes in one batch. Consequently, a subset of 154836 clones was identified which had ≤ 500 overlaps with other clones at 1e^-18^ (effectively removing clones with highly repeated fingerprint patterns) and this was used for subsequent map construction using LTCbeta 2.1. Unless otherwise stated, default settings were used. Initially, a network of significant clone overlaps was established with a cut of value p = 1x10^-18^ and Q clones were identified and removed using a cut-off value of 1x10^-28^. The remaining clones were then processed through the adaptive clustering procedure involving increasing the stringency of the cut-off in six equal steps of 3 orders of magnitude from 1x10^-18^ to 1x10^-33^ in order to identify the minimum stringency at which a contig satisfied the requirements of between 6 and 1000 clones per contig, inclusive, with a linear topology (see Frenkel *et al.* 2010). Following this first round of adaptive clustering, 15071 contigs still remained in 3 large clusters. These clones were extracted from the dataset and subjected to a second round of adaptive clustering which involved six equal steps of four orders of magnitude from 1x10^-18^ to 1x10^-42^. The contigs from these two adaptive clustering steps were combined to form the physical map version designated LTC-18(2s).

A second round of LTC physical maps were developed using the later version 1.4.6. For these maps the database of clone band sizes were processed in a single batch at each stringency level. For these maps, networks of significant clone overlaps were developed at the six different stringencies of 1x10^-12^, 1x10^-15^, 1x10^-18^, 1x10^-21^, 1x10^-24^ and 1x10^-27^ with clones with >500 significant overlaps excluded. Q clones were identified and excluded using cut-off values with stringencies ten orders of magnitude that of the initial cut-off value (*i.e.,* 1x10^-22^, 1x10^-25^, 1x10^-28^, 1x10^-31^, 1x10^-34^ and 1x10^-37^). Adaptive clustering was then implemented using 6 steps of 3 orders of magnitude of increasing stringency from the initial cut-off value, giving final cut-off values of 1x10^-30^, 1x10^-33^, 1x10^-36^, 1x10^-39^, 1x10^-42^ and 1x10^-45^, respectively. For each cut-off level, contigs of between 6 and 1000 clones per contig and with a linear topology were compiled to give the physical map versions designated LTC-12, LTC-15, LTC-18, LTC-21, LTC-24 and LTC-27.

Details of the physical maps are available in Supplementary Table S4, which can be downloaded from https://doi.org/10.20391/bb56e6d7-8913-4bd7-8167-2b7e4c01382b.
