# Supplementary material for: Integrating a newly developed BAC-based physical mapping resource for Lolium perenne with a genome-wide association study across a L. perenne European ecotype collection identifies genomic contexts associated with agriculturally important traits
Source: Ann Bot. 2019 Feb 2;123(6):977–92. doi: 10.1093/aob/mcy230 (PMC6589518; doi:10.1093/aob/mcy230)
Supplement: mcy230_suppl_Supplementary_Methods_S2 [file mcy230_suppl_supplementary_methods_s2.docx]

**Supplementary Methods S2.**

**Concatenation of BAC-derived contigs developed from the minimum tiling path for physical map version LTC-18(2s).**

The assemblies of BACs from the same LTC contig were concatenated. Contiguous BAC overlap sequences with perfect identify were collapsed using CD-HIT (v 4.6.3; Fu et al 2012 Bioinformatics). Later, mate-pairs libraries with insert lengths 3, 5 and 7 Kbp, built from whole genome DNA samples, were used to scaffold the sequences from each physical contig using SSPACE (v 2.0 basic; Boetzer et al 2011). By comparing BACs in the same FPC but different LTC contigs, we identified groups of physical contigs in close chromosomal regions. As before, these sequences were concatenated, collapsed with CD-HIT and scaffolded using mate-pairs libraries with insert lengths 20 and 23 Kbp. Finally, all the sequences were concatenated, collapsed and scaffolded, as before. Sequences under 5 Kbp were discarded. Finally, we did gap-filling with Pacbio long-reads using PBJelly (v. 2; English et al, 2012). All the mate-pair and Pacbio libraries were obtained from Aarnus University‘s genome assembly project (Byrne et al, 2015). These sequences were aligned to the actual reference (Byrne et al, 2015) using Minimap (v2, Li, 2018,). We retained every alignment longer than 5000 bp to produce a dotplot in R using dotPlotly (github.com/tpoorten/dotPlotly).

*Screening of MTP clones for potential cross-contamination.* In order to identify potential BAC-to-BAC sequence cross contamination, the database consisting of all contigs of ≥5kb was BLAST searched against itself using search parameters of word-size = 1000 and 100% sequence identity. Any contigs containing cross-matching sequences derived from BAC clone assemblies which were not predicted to be in the same physical map contigs were removed from the database. The resulting BAC sequence database, designated LpBAC5000, was used for anchoring GWAS markers.

**Boetzer M, Henkel CV, Jansen HJ, Butler D, Pirovano W.** **2011**. Scaffolding pre-assembled contigs using SSPACE. *Bioinformatics,* **27**: 578-579.

**English AC, Richards S, Han Y, Wang M, Vee V, Qu JX, Qin X, Muzny DM, Reid JG, Worley KC, Gibbs RA.** **2012**. Mind the Gap: Upgrading Genomes with Pacific Biosciences RS Long-Read Sequencing Technology. *Plos One:* e47768

**Fu LM, Niu BF, Zhu ZW, Wu ST, Li WZ.** **2012**. CD-HIT: accelerated for clustering the next-generation sequencing data. *Bioinformatics,* **28**: 3150-3152

**Li H.** **2018**. Minimap2: pairwise alignment for nucleotide sequences. *Bioinformatics*: bty191.
