# Supplementary material for: Integrating a newly developed BAC-based physical mapping resource for Lolium perenne with a genome-wide association study across a L. perenne European ecotype collection identifies genomic contexts associated with agriculturally important traits
Source: Ann Bot. 2019 Feb 2;123(6):977–92. doi: 10.1093/aob/mcy230 (PMC6589518; doi:10.1093/aob/mcy230)
Supplement: mcy230_suppl_Supplementary_Methods_S3 [file mcy230_suppl_supplementary_methods_s3.docx]

**Supplementary Methods S3**

***Construction of Superpools/Matrix pools.***

BAC pools for screening with PCR were constructed by Amplicon Express, Pullman, WA, USA. Briefly, the total number of 241920 BAC clones were arrayed into 630 384-well plates. DNA was extracted from all clones and the DNA from each batch of 7 consecutive plates was pooled to derive 90 superpools. Subsequently, for each superpool of 7 plates, 3-dimensional matrix pools were constructed consisting of 7 plate pools, 24 column pools and 16 row pools. To associate a PCR-based marker with particular BACs, the marker was first screened against the 90 matrix pools to identify which batches of 7 plates contained BACs positive for that marker sequence as detected by agarose gel electrophoresis. Subsequently, a subset of the matrix pools relating to the PCR-positive superpools were PCR-screened with the same marker and the pattern of PCR-positive plate, row and column pools could be used to identify individual BAC clones.

*Marker screening of Superpools and Matrix pools.* 1240 markers with known genetic/genomic positions were screened against the superpools and matrix pools using either agarose gel electrophoresis and primers and PCR conditions as described in King *et al*. (2013) or using markers for which assays were already available with KASP technology (LGC, Teddington, UK; Supplementary Table S2). Each marker was screened against a minimum of 3 sets of matrix pools relating to PCR-positive superpools and a physical map contig was considered to be assigned to a genetic/genomic position with confidence if at least two BACs from the same physical contig mapped to the same position.

**RAD-sequencing.**

A pilot RAD-sequencing and associated bionformatic analysis was carried out by Floragenex, Portland, OR, USA. The pilot study was developed to determine the effectiveness of RAD-sequencing based on *HindIII* restriction for assigning BAC clones to genetic positions. Both pooled DNA from 10 genotypes and a set of 8 genotypically contrasting genotypes from an F2 *L. perenne* mapping family (Turner *et al.* 2006) were RAD sequenced using *HindIII* restriction. Resulting sequences were aligned to the related *HindIII* BAC-end sequence (BES) library (described below) and potential SNP polymorphisms relating to BES in the F2 mapping family identified using the in-house Floragenex bioinformatic pipeline. A subset of 732 potential SNP markers were then screened across 96 genotypes of the F2 mapping family using Sequenom MassARRAY technology at the Wellcome Trust Centre for Human Genetics, Oxford, UK, and validated by genetic mapping in the F2 family
