# Supplementary material for: Integrating a newly developed BAC-based physical mapping resource for Lolium perenne with a genome-wide association study across a L. perenne European ecotype collection identifies genomic contexts associated with agriculturally important traits
Source: Ann Bot. 2019 Feb 2;123(6):977–92. doi: 10.1093/aob/mcy230 (PMC6589518; doi:10.1093/aob/mcy230)
Supplement: mcy230_suppl_Supplementary_Methods_S4-S5 [file mcy230_suppl_supplementary_methods_s4-s5.docx]

**Supplementary Methods S4 - Field Measurements**

The 716 genotypes described in Blackmore et al. (2015) and in Supplementary Table S1 were grown as spaced plants in 3 replicated blocks at a field site in Aberystwyth, UK. Plants were hand planted with 60cm spacing between plants with guard plants surrounding each replicated block. Management of plants followed standard procedures with fertiliser 23N:4P:13K with 7% sulphur applied at 80kgs in spring and 80kgs before the second cut in late summer. A further 50kgs was applied before any other times the plants were cut back. The plants were kept clean with a combination of intra row spraying with glyphosate weed killer and hand weeding.

Field-based measurements were conducted according to standard practices used in perennial ryegrass national variety assessments.

- Plant establishment was scored as 1-9 :-with 1= dead, 9 = very good establishment
- Plant width scored 1-9 :-1= very narrow, 9 = large plant
- Leaf width scored 1-9:- 1 very narrow leaves, 9 broad leaves.
- Vegetative biomass scored when plants were heading visual score 1-9 where 1 = very low biomass, 9 = high biomass
- Heading date was recorded when 3 inflorescences on a plant emerged from flag leaf.
- Plant regrowth scored in July, 5 weeks after cutting back and in September, 4 weeks after cutting back 1-9:- 1 = no regrowth, 9 = very good regrowth
- Stem rust was measured on a 1-9 scale with 1= resistant 9 highly susceptible
- Spring growth scored 1^st^ May scored 1-9 1 =poor growth 9= good growth
- Dry weight, plants cut back to 5cms and all plant material dried overnight at 80^0^C.

**Supplementary Methods S5– Analytical Chemistry**

**Spectrum Collection**

Dried and ground samples of dried and milled grass were scanned in small ring cups of 36 mm diameter using a NIRSystems model 6500 near-infrared scanning monochromator (FOSS NIRSystems Inc., Laurel, MD). The samples were scanned (average of 32 successive scans) over the wavelength range from 400 to 2498 nm at 2 nm intervals using WinISI II 1.04a (Infrasoft International LLC, State College, PA) software.

**Population and Sample Selection**

Samples representative of the population were selected based on Mahalanobis H (Global H) and neighbourhood H (NH) distances as described by Shenk and Westerhaus, 1991a,b.

**Calibration and validation Procedures**

During calibration development only spectral data from 1100–2498 nm was used (a total of 700 data points for each sample). Calibration models were developed as described by Sanderson *et al*., (1997) for water soluble carbohydrate (WSC), nitrogen (N), dry matter digestibility (DMD). Standard normal variate (SNV) and de-trending transformations were used (Barnes *et al* , 1989). During development of mPLS equations, cross validation was used to avoid overfitting and to study the robustness of the calibration models and two outlier elimination passes were performed. The final equations were selected on the basis of minimising SECV and increasing R^2^_CV_. The methods used to select samples for equation update are described in Shenk & Westerhaus (1991a,b).

**Reference methods**

WSC was extracted with cold water and analysed using the method described by Thomas (1977). The released sugars determined spectrophotometrically as the blue/green complex formed when carbohydrates are heated with anthrone in sulphuric acid. Nitrogen (N) was analysed by a rapid combustion method using a LECO FP-428 analyser (LECO Corp., St. Joseph, MI). DMD was determined using the two-stage pepsin-cellulase in-vitro method described by Jones & Hayward (1975). Dry matter solubility was then used to estimate DMD from a regression equation derived from samples of known in vivo digestibility.

ADF (ash free) was determined according to the method described by Van Soest et al (1991) using the Fibrecap system (Kitcherside et al 2000). NDF (ash free) was determined according to the method described by Van Soest et al (1991) without the use of termamyl or sodium sulphite using the Fibrecap system (Kitcherside et al 2000).

**References:**

Barnes, R.J., Dhanoa, M.S. and Lister, S.J. 1989 Standard normal variate transformation and de-trending of near-infrared diffuse reflectance spectra. Applied Spectroscopy **43**: 772-777.

Jones, D I H and Hayward, M V (1975) The effect of pepsin pre-treatment of herbage on the prediction of dry matter digestibility for solubility in fungal cellulase solutions. *J. Sci. Fd. Agric.,* **26:** 711-718.

Kitcherside, M.A., Glen, E.F. & Webster A.J.F. (2000) Fibrecap: an improved method for the rapid analysis of fibre in feeding stuffs. Anim. Fd. Sci. Technol. 86, 125 – 132.

Sanderson, R., Lister, S.J., Dhanoa, M.S., Barnes, R.J. and Thomas, C. Use of near infrared reflectance spectroscopy to predict and compare the composition of carcass samples from young steers. Animal Science, 1997, **65**:45-54.

Shenk, J.S. and Westerhaus, M.O. (1991a) Population definition, sample selection, and calibration procedures for near infrared spectroscopy. *Crop Science*, **31**: 469-474.

Shenk, J.S. and Westerhaus, M.O. (1991b) NIRS analysis of agricultural products using population structuring and modified PLS regression. *Crop Science*, **31**: 1548-1555.

Thomas, T.A. (1977) An automated Procedure for the Determination of Soluble Carbohydrates in Herbage. *J. Sci. Fd Agric.,* **28**: 639-642

Van Soest, P.J., Robertson, J.B., and Lewis, B.A. (1991) Methods for Dietary Fiber, Neutral Detergent Fiber, and Nonstarch Polysaccharides in Relation to Animal Nutrition. J. Dairy Sci., 74: 3583-3597.
