# Supplementary material for: Integrating a newly developed BAC-based physical mapping resource for Lolium perenne with a genome-wide association study across a L. perenne European ecotype collection identifies genomic contexts associated with agriculturally important traits
Source: Ann Bot. 2019 Feb 2;123(6):977–92. doi: 10.1093/aob/mcy230 (PMC6589518; doi:10.1093/aob/mcy230)
Supplement: mcy230_suppl_Supplementary_Results_S1 [file mcy230_suppl_supplementary_results_s1.docx]

**Supplementary Results S1.**

**Concatenation of BAC assemblies and alignment with assembly of Byrne et al (2015).**

After merging the individual BAC assemblies, we obtained 200387 sequences with an average sequence length of 19.7 Kbp. This assembly consisted of 3.94 Gb and included 21.5 % ambiguous nucleotides (Ns), and had a N50 of 29.78 Kb.. Approximately half of the expected genome size (2.4 Gb) was recovered in the 15,558 sequences over 50 Kb (Supplementary Fig. S4). A total of 175,632 of the sequences (87,6 %) showed an alignment of >1000 nucleotides perfect matches on the existing genome reference (Byrne et al 2015). We filtered 335450 alignments longer than 5 Kb, which comprised 136,834 unique sequences, and anchored them on the published genome (Supplementary Fig. S5). The individual BAC assemblies, as well as the merged assembly, are deposited online in SRA under Bioproject PRJNA475227; BioSample:SAMN09382314; SRA Sample:SRS3412769; SRA Study:SRP150420


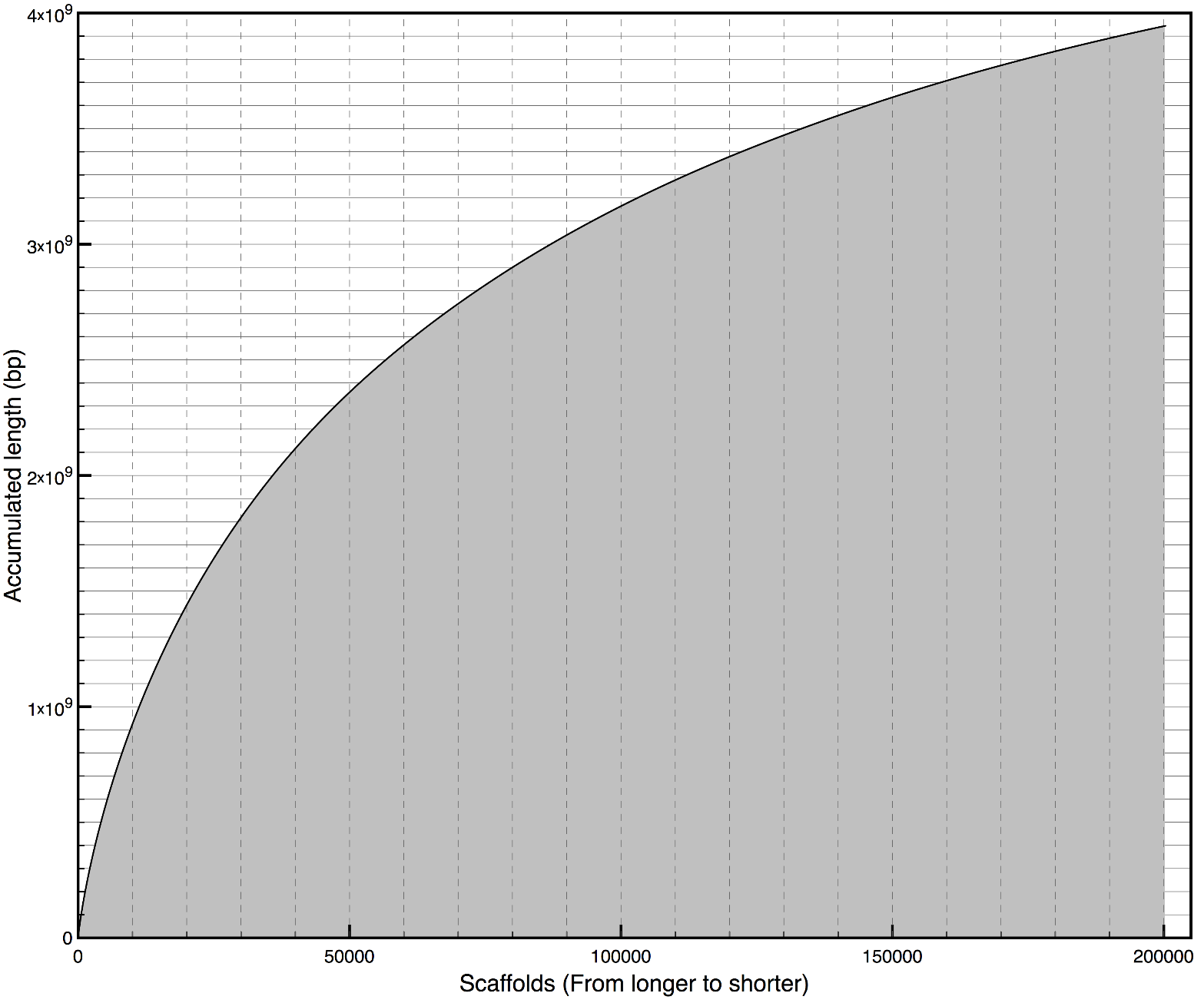


**Supplementary Fig. S4.** Accumulated contig sequence lengths derived from BACs in the minimum tiling path for physical map version LTC-18(2s).


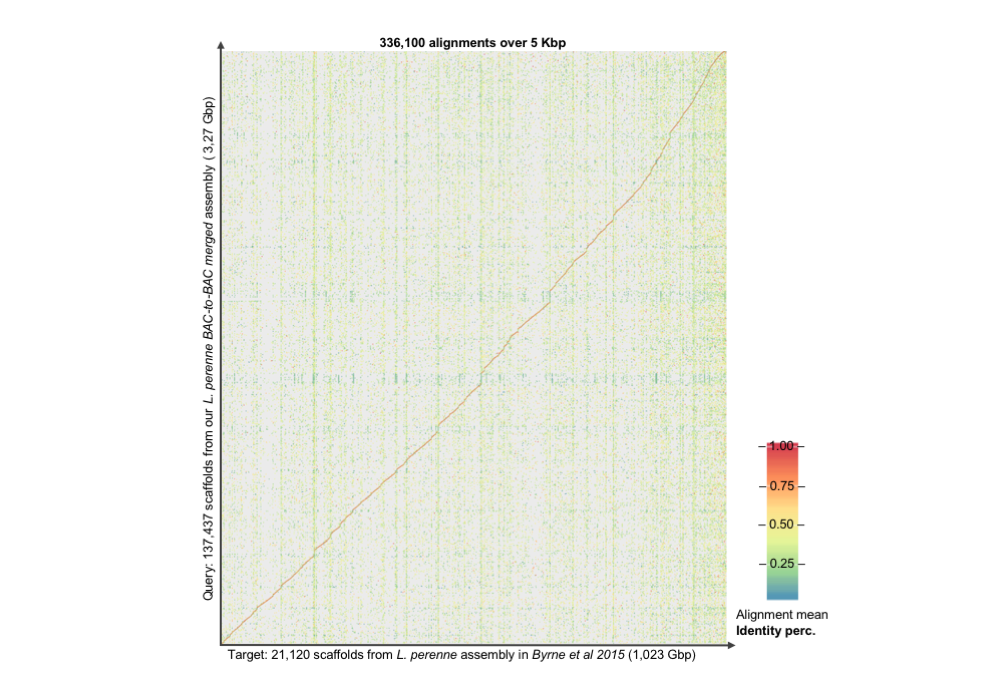


**Supplementary Fig. S5.** Alignment of the concatenated sequence assembly developed from BACs in the minimum tiling path for physical map version LTC-18(2s) with the published *L. perenne* draft genome of Byrne et al, 2015,

**Removal of potential cross-contaminating sequences.**

BLAST searches of this sequence database, containing the contigs derived from these BACs, against itself identified 32960 contigs as containing identical sequence stretches located in BACs not from the same physical contigs according to the search parameters of word-size = 1000 and 100% sequence identity. Because these sequence stretches might represent cross-contaminating reads, these reads and the associated contigs were removed to derive the LpBAC5000 database used in subsequent marker screening.

**RAD sequencing pilot study**

RAD sequencing followed by alignment to BES identified c. 26k potential SNP polymorphisms aligning to c. 21k BES from the pooled F2 family DNA, and c. 32k potential SNP polymorphisms aligning to c. 8.5k BES from the comparison of the 8 genotypes (see Supplementary Methods S4). Based on polymorphism frequency across aligned sequences, high quality flanking sequences, the presence of the identified BACs in physical contigs and distribution across different contigs, 443 SNPs from the pooled DNA predictions and 289 SNPs from the 8 genotype comparisons were scored across the F2 family and genetically mapped within the existing dataset. The conversion rate from predicted SNPs to successfully mapped BES was c. 30% for pooled DNA and c. 42% for the comparison across genotypes.
