# Supplementary material for: Integrating a newly developed BAC-based physical mapping resource for Lolium perenne with a genome-wide association study across a L. perenne European ecotype collection identifies genomic contexts associated with agriculturally important traits
Source: Ann Bot. 2019 Feb 2;123(6):977–92. doi: 10.1093/aob/mcy230 (PMC6589518; doi:10.1093/aob/mcy230)
Supplement: mcy230_suppl_Supplementary_Table_S3 [file mcy230_suppl_supplementary_table_s3.docx]

| **Supplementary Table S3.** Summary statistics for BAC library production and fingerprinting | | | | | | | | |  |
| --- | --- | --- | --- | --- | --- | --- | --- | --- | --- |
| **BAC Library** | | | | **BAC-end sequence** | | | **BAC HICF** | |  |
| **Name** | **Enzyme** | **Clones** | **Av. insert size (kb)** | **Sequenced clones** | **%** | **Avg. size (bp)** | **Fingerprinted clones** | **%** | |
| LPP_Ba | *HindIII* | 120960 | 134 | 196170 | 82 | 558 | 102962 | 85 | |
| LPP_Bb | *BstYI* | 120960 | 120 | 195094 | 82 | 514 | 109961 | 90 | |
| Total |  | 241920 | 127 | 391264 | 82 | 536 | 212923 | 88 | |

| **Supplementary Table S5A.** LpBAC5000 sequence size range per contig | | |
| --- | --- | --- |
| **Contig size range (bp)** | **Number of contigs** | **bp(Mb)** |
| 5000-9999 | 109953 | 778 |
| 10000-14999 | 44161 | 541 |
| 15000-19999 | 22792 | 393 |
| 20000-49999 | 29513 | 829 |
| >50000 | 1539 | 93 |
| **Total** | 207958 | 2634 |
|  |  |  |
| **Supplementary Table S5B.** LpBAC5000 sequence size range per BAC | | |
| **Sequence size range/BAC (kbp)** | **Number of BACs** | **%** |
| 5-20 | 1338 | 4 |
| 20-30 | 1193 | 5.56 |
| 30-40 | 1652 | 4.93 |
| 40-50 | 2262 | 6.75 |
| 50-60 | 2836 | 8.47 |
| 60-70 | 3629 | 10.84 |
| 70-80 | 3876 | 11.58 |
| 80-90 | 4075 | 12.17 |
| 90-100 | 3925 | 11.72 |
| 100-110 | 3220 | 9.62 |
| 110-120 | 2331 | 6.96 |
| 120-130 | 1521 | 4.54 |
| 130-140 | 895 | 2.67 |
| 140-150 | 426 | 1.27 |
| 150-160 | 194 | 0.58 |
| 160-170 | 74 | 0.22 |
| 170-180 | 30 | 0.09 |
| 180-190 | 3 | 0.01 |
| **Total** | 33480 |  |

| **Supplementary Table S6.** Estimates of genome coverage within the physical maps. | | | |
| --- | --- | --- | --- |
| **Physical map** | **Total HICF bands** | **Gb coverage (33480 clones)^1^** | **Gb coverage**  **(39202 clones) ^2^** |
| **FPC-1.02** | 2517826 | 1.73 | 2.04 |
| **FPC-1.03** | 2294198 | 1.57 | 1.85 |
| **LTC-12** | 2292434 | 1.57 | 1.85 |
| **LTC-15** | 2425891 | 1.66 | 1.95 |
| **LTC-18** | 2595424 | 1.78 | 2.09 |
| **LTC-21** | 2800358 | 1.92 | 2.26 |
| **LTC-24** | 2977429 | 2.04 | 2.4 |
| **LTC-27** | 3084672 | 2.11 | 2.5 |
| **LTC-18(2s)** | 2576841 | 1.76 | 2.07 |
| ^1^ (Total sequence length per clone/HICF bands per clone) x total HICF bands in physical map | | | |
| ^2^ Gb coverage in ^1^ x 1.18 (correction factor for only 85% of MTP clones sequenced) | | | |
